# Landscape of mobile genetic elements and their antibiotic resistance cargo in prokaryotic genomes

**DOI:** 10.1093/nar/gkac163

**Published:** 2022-03-22

**Authors:** Supriya Khedkar, Georgy Smyshlyaev, Ivica Letunic, Oleksandr M Maistrenko, Luis Pedro Coelho, Askarbek Orakov, Sofia K Forslund, Falk Hildebrand, Mechthild Luetge, Thomas S B Schmidt, Orsolya Barabas, Peer Bork

**Affiliations:** European Molecular Biology Laboratory, Structural and Computational Biology Unit, 69117 Heidelberg, Germany; European Molecular Biology Laboratory, Structural and Computational Biology Unit, 69117 Heidelberg, Germany; Department of Molecular Biology, University of Geneva, 1211 Geneva, Switzerland; Biobyte solutions GmbH, Bothestr 142, 69117 Heidelberg, Germany; European Molecular Biology Laboratory, Structural and Computational Biology Unit, 69117 Heidelberg, Germany; Institute of Science and Technology for Brain-Inspired Intelligence, Fudan University, Shanghai 200433, China; European Molecular Biology Laboratory, Structural and Computational Biology Unit, 69117 Heidelberg, Germany; European Molecular Biology Laboratory, Structural and Computational Biology Unit, 69117 Heidelberg, Germany; Max Delbrück Centre for Molecular Medicine, Berlin, Germany; Experimental and Clinical Research Center, Charité-Universitätsmedizin and Max-Delbrück Center, Berlin, Germany; Charité – Universitätsmedizin Berlin, Berlin, Germany; European Molecular Biology Laboratory, Structural and Computational Biology Unit, 69117 Heidelberg, Germany; European Molecular Biology Laboratory, Structural and Computational Biology Unit, 69117 Heidelberg, Germany; European Molecular Biology Laboratory, Structural and Computational Biology Unit, 69117 Heidelberg, Germany; European Molecular Biology Laboratory, Structural and Computational Biology Unit, 69117 Heidelberg, Germany; Department of Molecular Biology, University of Geneva, 1211 Geneva, Switzerland; European Molecular Biology Laboratory, Structural and Computational Biology Unit, 69117 Heidelberg, Germany; Max Delbrück Centre for Molecular Medicine, Berlin, Germany; Department of Bioinformatics, Biocenter, University of Würzburg, Würzburg, Germany; Yonsei Frontier Lab (YFL), Yonsei University, Seoul 03722, South Korea

## Abstract

Prokaryotic Mobile Genetic Elements (MGEs) such as transposons, integrons, phages and plasmids, play important roles in prokaryotic evolution and in the dispersal of cargo functions like antibiotic resistance. However, each of these MGE types is usually annotated and analysed individually, hampering a global understanding of phylogenetic and environmental patterns of MGE dispersal. We thus developed a computational framework that captures diverse MGE types, their cargos and MGE-mediated horizontal transfer events, using recombinases as ubiquitous MGE marker genes and pangenome information for MGE boundary estimation. Applied to ∼84k genomes with habitat annotation, we mapped 2.8 million MGE-specific recombinases to six operational MGE types, which together contain on average 13% of all the genes in a genome. Transposable elements (TEs) dominated across all taxa (∼1.7 million occurrences), outnumbering phages and phage-like elements (<0.4 million). We recorded numerous MGE-mediated horizontal transfer events across diverse phyla and habitats involving all MGE types, disentangled and quantified the extent of hitchhiking of TEs (17%) and integrons (63%) with other MGE categories, and established TEs as dominant carriers of antibiotic resistance genes. We integrated all these findings into a resource (proMGE.embl.de), which should facilitate future studies on the large mobile part of genomes and its horizontal dispersal.

## INTRODUCTION

Prokaryotic Mobile Genetic Elements (MGEs) can transfer horizontally and play an important role in prokaryotic species evolution as they often give the host fitness advantages e.g. in bacterial survival, species diversification and niche expansion (1–3), by transferring adaptive functions such as antibiotic resistance (4). The latter, when acquired by human pathogens, has risen to one of the major challenges in public health (5). Hence, to understand global patterns of MGE-driven emergence of multi-drug resistance, the general dispersal of molecular functions and prokaryotic species evolution, one needs a cohesive and comparative quantification of different MGE types, their adaptive functions as well as their horizontal transfer potential.

However, MGE is an umbrella term for a number of structurally and mechanistically distinct types of elements. Plasmids (replicons that transfer between cells via conjugation (6), up to 2.5 Mb in length), Integrative Conjugative Elements (ICEs, which integrate into the host genome and carry a functional conjugation system for inter-cellular transfer (7), ∼18–500 kb in length) and phages (forming viral particles that infect a prokaryotic cell, replicating within it and are transferred between the cells via transduction (8), ∼11–500 kb in length) are capable of inter-cellular transfer. Insertion sequences (IS, elements carry only a transposase gene (9), ∼2.5 kb in length) and, transposons (elements that carry transposase and dispensable cargo genes (10), ∼5 kb in length) and integrons (gene acquisition systems that are immobile without other MGEs (11), several kb in length) depend on other MGEs for inter-cellular transfer. A wide variety of methods, tools and databases catalogue the different experimentally studied MGEs, usually per type (12,13). Widely used examples of databases are ISfinder (10) (for IS elements and transposons), ICEberg (14) (for ICEs and their derivatives) and ACLAME (15) (for IS elements, transposons, phages, plasmids). In addition, more generic annotation resources such as PFAM (16), EggNOG (17) and KEGG (18) offer broad family level annotations of certain MGE-associated genes. Altogether, there are many tools to annotate MGE genes as well as to estimate MGE boundaries. However, neither a single method nor a combination of methods allows a unified annotation and classification of all MGE types for comparative analyses.

Despite their differences, all MGEs that impact the host genome share a function-based common denominator, namely a gene coding for an enzyme (either a recombinase, transposase or nuclease, hereafter broadly referred to as recombinase). These recombinases are responsible for the integration and excision of IS elements, transposons, ICEs and phages, gene cassette acquisition of integrons (11), as well as for separation and segregation of newly replicated plasmids and phage chromosomes (19–21). Although the recombinases can belong to very different non-homologous protein families, the biochemical mechanisms and substrate specificities of respective recombinase families are still MGE type- and subtype-specific (see Supplementary Table S1). Taking this into account, we developed a unifying framework by utilising recombinase subfamilies as essential MGE marker genes and pangenome information as the basis for MGE boundary estimation. Applied to 84,022 high quality genomes (22), we identified 2.8 Mio MGE recombinases and, using a knowledge based approach assigned them to six operational MGE categories, based on known MGE types. This allowed us to: (i) comparatively quantify phylogenetic and environmental prevalence of MGEs, (ii) provide a lower limit for MGE-mediated distant horizontal gene transfer events across clades and habitats and (iii) show the potential of our framework for characterising MGE cargos such as antibiotic resistance genes, to reveal that the vast majority of resistance genes reside in transposable elements. To facilitate MGE research in prokaryotes, we integrated all the respective data and results into a public resource (proMGE.embl.de).

## MATERIALS AND METHODS

### HMM building and calibration

We built supervised HMM profiles for serine, HUH-Y1 and Y2 and Cas1 solo recombinases using known protein sequences belonging to the respective recombinase families:

A. For serine and HUH recombinase we performed an hmmsearch (HMMER3.1b2) against ICEberg database (14) (downloaded in November 2017) using Pfam31.0 HMMs (for serine recombinase - PF00239 and HUH recombinase as listed in Supplementary Table S1) with the option --cut_ga. We further supplemented the hmmsearch besthits with protein sequences of serine recombinases (belonging to IS607 and Tn3 family transposons) and HUH recombinase (HUH-Y1 and HUH-Y2) from ISfinder database (10) and protein sequences of cas1 solo recombinases from (23).

B. For each recombinase family, we performed the following analysis independently as shown in Supplementary Figure S1: we aligned sequences using Clustal Omega 1.2.4 (24) and built a phylogenetic tree using FastTree 2.1.10 SSE3 to identify monophyletic clusters based on tree-topology. For each of these clusters we built an HMM using HMMER 3.1b2 with default parameters and defined a gathering threshold based on hmmsearch best hits of all protein sequences used to build the recombinase family phylogenetic tree as described by Pfam (16). To increase the sensitivity of the built HMMs we followed the following steps iteratively for each recombinase cluster (subfamily):

Step 1. We performed hmmsearch using the newly built HMMs with the --cut_ga option enabled against proGenomes1(25), ACLAME (15) and ICEberg database (14).

Step 2. We combined the protein sequences of best hits from Step 1 with the known protein sequences that formed the cluster (subfamily) described above.

Step 3. We removed redundant protein sequences by performing CD-HIT version 4.6.8 clustering using the parameters -c 1.00 -n 5 -T 8 -d 0 and built HMM of the obtained non-redundant protein sequences using HMMER 3.1b2 with default parameters.

Step 4. We performed 10-fold cross validation to determine the gathering threshold of an HMM, using a training set consisting of true positive sequences from Step 3 and true negative sequences from other subfamilies belonging to the same recombinase family.

We computed the gathering threshold for each fold using the pROC package in R (26) which optimises for specificity and sensitivity in each case. However, for cases where the ROC derived threshold was higher than the bit score of known true positive, the highest bit score of known true positive was considered.

Step 5. Median of gathering thresholds of folds with Matthews Correlation Coefficient (27) MCC > 0.8 was considered as gathering threshold for the HMM.

Step 6. An hmmsearch using the newly built HMMs with the --cut_ga option enabled was performed against the proGenomes1, ACLAME and ICEberg databases.

Steps 1–6 were iterated until a. The number of best hits in Step 6 were fewer than the number of best hits in the previous iteration; b. The hmmsearch did not retain all the known original sequences used to build the recombinase subfamily cluster; c. The hmmsearch best hits of different subfamilies showed an overlap; d. False positives were detected based on spurious protein annotations. EggNOG (17) annotations were considered for proteins from proGenomes1 database and protein descriptions were considered for proteins from ACLAME and ICEberg databases.

If an iteration met any of these four conditions the HMM from the previous (i – 1) iteration was deemed final and used further.

### Recombinase census: annotation and sensitivity analysis

**Figure 1.**
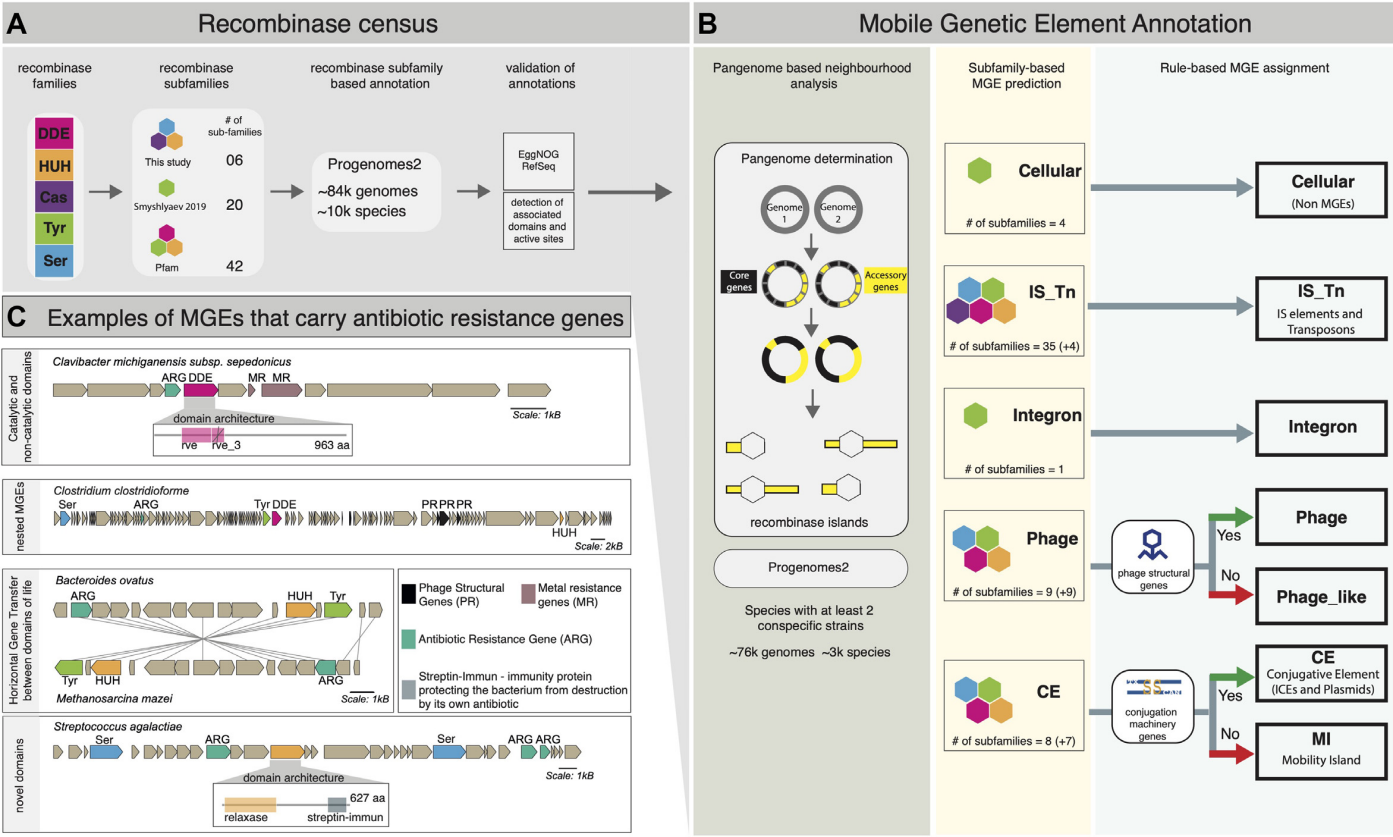
Prokaryotic MGE identification workflow and cargo analysis. (**A**) The five major recombinase families were annotated in proGenomes2 (22) based on subfamily-level HMMs, validated based on EggNOG (17) and Refseq (55) annotations, as well as by the presence of known associated domains and catalytic site residues. (**B**) MGE assignment of identified recombinases into six major MGE categories and distinction from cellular recombinases using i) pangenome-based boundary estimation and neighbourhood analysis of core genes (black) and accessory genes (yellow) in 76k genomes, ii) association of MGE types with recombinase subfamilies and iii) rules requiring the presence of MGE type-specific accessory genes. (**C**) Selected examples of identified MGEs carrying ARG (Antibiotic Resistance Gene) cargo, illustrating different aspects of novelty revealed by in-depth analysis. Catalytic and non-catalytic domains: presence of two recombinase domains one of which (‘rve_3’, crossed) putatively lacks the active site implies functional diversification. Nested MGEs: co-localisation of multiple recombinases belonging to diverse families on recombinase islands. Horizontal Gene Transfer (HGT) between domains of life: the recombinase and almost all neighbouring genes within the MGE show high sequence similarity (> 95% nucleotide sequence identity) between the bacterium *Bacteroides ovatus*and the archaeon *Methanosarcina mazei*, implying HGT between domains of life. Novel domain associations: a ‘Streptin-Immun’ domain within a relaxase, confers the host with antibiotic auto-immunity (53).

We used six HMMs built as described above and 62 additional HMMs from Pfam31.0 version and (28) as described in Supplementary Table S1 and Figure 1A to annotate recombinases within proGenomes2 (22) using hmmsearch with --cut_ga option using HMMER 3.1b2.

- Comparison to EggNOG annotations: To compute the recall statistics of our annotations we built a database of probable true positive hits based on text mining of proGenomes2 EggNOG annotations. We extracted the proGenomes2 annotations of all proteins that matched the term recombinase, integrase, transposase, tyrosine recombinase, serine recombinase, resolvase, relaxase, insertions sequence, IS, DDE and filtered out hits which matched invertase, replication protein, DNA repair, DNA binding, homologous recombination, putative for example. We filtered out hits which were annotated as transposase/integrase and showed absence of any domains or absence of transposase/integrase catalytic domain as determined by independent PfamA search (similar procedure was followed for detecting recombinases among RefSeq proteins downloaded in September 2020 ftp://ftp.ncbi.nlm.nih.gov/genomes/all/GCF/) (Pfam31.0) (these filtered out proteins that contained transposon and integrase specific HTH and associated domains (but no known catalytic domains), but these were annotated as transposase within Pfam giving rise to their annotation as transposase by EggNOG (17)). Rest of the filtered-out proteins included viral exonuclease proteins that act on single stranded DNA (YqaJ), proteins annotated as relaxases which contain a helicase domain but not a recombinase domain. In total, we obtained ∼2.3 million proteins within proGenomes2 that potentially belonged to dsDNA based recombinases of mobile element origin only.
- Analysis of recombinase active-site residues: Information about active site residues of different recombinase families DDE recombinase, HUH recombinase, serine recombinase, tyrosine recombinase and cas1 solo was obtained. In the 2.9 million proteins annotated in proGenomes2 we looked for presence and positional conservation of active site residues in all the catalytic recombinase domains present within a protein. For recombinases where more than one residue was part of the active site, we determined whether the pairwise inter-residue distance was conserved, in accordance with their spatial distribution reported in literature (serine recombinase: S10 (29,30); DDE (31): DD 20–100, DE > 100; cas1 solo: DH < 20, DE < 87; HUH (32): HH = 2 (A/D/N), HY 50–160; tyrosine recombinase (33): YR 22–42, YH < 50, YK 95–115). For proteins with multiple catalytic domains, the protein was classified as active if at least one of the catalytic domains showed presence and positional conservation as described above.
- Recombinase domain association analysis: We performed an PfamA (Pfam 31.0) hmmsearch against all the proteins in proGenomes2 with the --cut_ga and --domtblout options enabled using HMMER 3.1b2 and determined their domain composition. For each of the 68 HMMs used in this study, we determined the domains associated with these by performing one sided Fisher's exact test. Significant domain associations were determined after correcting for multiple testing using the Bonferroni method.

### Mobile genetic element annotation

To define core and accessory genes, species pangenomes were derived from gene clusters reported in GMGC (http://gmgc.embl.de/) at >95% nucleotide identity and presence in 95% of the conspecific strains in a species, where species with at least two conspecific strains were considered. We described accessory gene regions (islands) of a bacterial genome by stitching together contiguous stretches of accessory genes until a core gene or a contig boundary was encountered, and vice versa for core gene regions (islands). We mapped our 68 HMM derived recombinase protein annotations to all the islands. We referred to these recombinases containing islands as recombinase islands (both core and accessory). In total, 75.7% of our MGE recombinase island boundaries where well within both contig boundaries, the rest had at least one end of the MGE recombinase island as contig boundary.

We then mapped phage structural gene annotations from EggNOG (17) and annotated genes involved in conjugation using TXSscan (34) to the recombinase islands. As shown in Figure 1B and Supplementary Figure S7 we first made subfamily based MGE prediction of the recombinase containing islands based on known association of recombinase subfamily with MGE type (23,28,35–37). For recombinase subfamilies which were reported as associated with more than one MGE type (Supplementary Table S1, Figure 1 and Supplementary figure S5A) we made MGE assignment by following a rule-based system (Supplementary Table S1) that supplemented the subfamily based MGE prediction with additional evidence about presence or absence of phage structural genes/genes involved in conjugation, within the boundaries of recombinase islands. For recombinase islands with multiple recombinases belonging to same or different MGE type referred to as nested recombinase islands, the MGE boundary corresponds to the entire recombinase island boundary. The boundaries of individual MGEs within a nested recombinase are not disentangled. Example 1: For recombinase island containing more than one recombinase belonging to transposable elements, the MGE boundary is same as that of the recombinase island. The transposable element count for this recombinase island is equal to the number recombinases associated with transposable elements. Example 2: For recombinase island containing more than one recombinase of which one is a phage recombinase and the other two are recombinases associated with transposable elements again the MGE boundary is same as the boundary for the recombinase island. In this case, the transposable element count will be two and phage count will be one (provided phage structural genes are found in the recombinase island else phage_like count will be one)

### Marker gene based phylogenetic tree

Marker genes were predicted and selected using fetchMG (38). We aligned protein sequences of each of the 40 universal marker genes (39) using Muscle 3.8.31 (40). Each marker gene alignment was concatenated using a custom script https://github.com/nylander/catfasta2phyml following the protocols of Ciccarelli *et al.* (39). When a marker gene was missing in the genome a gap was introduced into the alignment (38). The phylogenetic tree was built using the concatenated alignment of each marker gene with the FastTree 2.1.11 (41) tree-builder.

### MGE: taxonomic class association analysis

Average MGE counts per species per taxonomic class were computed and one-sided Wilcox test was performed to determine association of each MGE type with each taxonomic class. The obtained *P*-values were corrected for multiple testing using Bonferonni method and *P*-values <0.05 were considered for determining significant association.

### Comparison to ISEScan and PHASTER predictions

Twenty-three genomes belonging to each of the 23 taxonomic classes in Figure 3A were sampled for this analysis. For phage predictions, the online version of PHASTER (42), which relies on sequence similarity to a phage protein database was rendered and phage predictions with score >70 were considered for comparison with phage and phage_like MGE categories. For transposable element predictions, a standalone version of ISEScan (43) which combines uncalibrated HMM searches of transposases derived from the ACLAME database of mobile element proteins (15) and terminal inverted repeat detection for transposable elements boundary prediction was used. All the predicted transposable elements were considered for comparison. Variation in MGE boundary lengths was calculated by subtracting length of ISEScan or PHASTER predicted MGE with lengths of MGE predicted in this study (Supplementary Table S3).

### Detection of HGT events

To infer HGT of recombinases, all the 2.9 million recombinases were clustered using CDHIT version 4.6.8 with cd-hit-est command and parameters -c 0.95 -n 11 -s 1 -l 100 -g1 -G1 (95% identity threshold) and -c 1 -n 11 -s 1 -l 100 -g1 -G1 (100% identity threshold/identical sequences). As our species definitions rely on a marker gene based approach (38), which partially differs from NCBI taxonomy, only a subset of 63k of the 76k genomes with taxonomic hierarchies consistent with NCBI were considered. Few genomes (*n* = 154) were filtered out using GUNC (44) https://github.com/grp-bork/gunc if their clade separation score (CSS) was >0.6 and contamination portion was >5%. MGE recombinases belonging to different genomes that clustered together were considered to be horizontally transferred between the respective genomes they were found in if they belonged to different taxonomic families given their taxonomies based on specI (38) and NCBI taxonomy. HGT events were classified at different taxonomic levels depending on whether they belonged to the same family, order, class, phylum and kingdom/domains of life. We calculated HGT events at family level and above. HGT events were counted as the total number of paired combinations of taxa where the MGE recombinase cluster was found. In the minority of the cases where it was not possible to resolve MGE assignments based on recombinase due to nested MGEs they were flagged as unresolved and excluded from any further analysis.

We ensured that the 13 kb HGT event between domains of life represented in Figure 1C was not a technical artefact based on (a) the observation that neither of the genomes were chimeric as analysed using GUNC (44); (b) both the genomes came from independent studies as reported during submission of sequencing data on NCBI; c) the recurrent presence of same MGE recombinase clusters across the two domains of life (e.g. in seven archaeal genomes belonging to phylum Euryarchaeota and five bacterial genomes belonging to Bacteroidetes (1 genome) and Firmicutes (4 genomes) phyla)

### Nested MGE calculations for HGT analysis

To determine the nestedness of transposons and integrons with other MGE categories in our HGT dataset, we determined the number of times each transposon/integron recombinase or recombinase cluster (see above) that co-occurred with recombinases from other MGE categories on a recombinase island. In the case of HGT events, we only considered the cases where two recombinase clusters (one from transposons/integrons and another from other MGE categories) co-occurred on a recombinase island across two distinct taxa. In each case, we then determined the MGE category of the second recombinase.

### Habitat data and analysis

As habitat assignment of cultivated species is often ambiguous, we used habitat information for genes from the Global Microbial Gene Catalogue (GMGC) derived from metagenomic studies (http://gmgc.embl.de/). This included following habitat categories: human-gut, cat-gut, dog-gut, pig-gut, mouse-gut, human-oral, human-nose, human-skin, human-vagina, built-environment, marine, freshwater, soil and wastewater. In cases where a gene was associated with multiple habitats, all the corresponding habitats were considered. MGE counts for each category were also normalised by the total number of MGEs belonging to that category found in the habitat.

### Antibiotic resistance genes (ARG) annotations and enrichment analysis

Antimicrobial resistance gene annotations were obtained using a previously described workflow (22) with several modifications. In short, per-gene antimicrobial resistance potential was inferred via both the Comprehensive Antibiotics Resistance Database (CARD) Resistance Gene Identifier (RGI) tool (45) v3.0.1 in ‘strict’ mode, and ResFams (46) v1.2.2 using HMMER3 (47) v3.1b2 ‘hmmsearch’ with the ‘--cut_ga’ option for model-specific gathering thresholds. The resulting annotations were then consolidated via the Antibiotics Resistance Ontology (ARO) (https://github.com/arpcard/aro) with additional manually curated matching of terms, giving preference to CARD RGI calls. Annotations were additionally filtered by removing hits to ResFams models RF0038, RF0039, RF0058, RF0085 and RF0086 which produced almost exclusively spurious hits among manual inspection. Antimicrobial resistance type, mechanism and antimicrobial drugs to which resistance was putatively conferred were derived from ARO terms.

For each MGE category we computed the average number of ARGs per 1000 genes based on MGEs that showed presence of at least one ARG and the total number of protein coding genes within the element. For each MGE category we determined the total number of ARGs and determined their enrichment using one sided Fisher's exact test. Statistical significance was determined after correcting for multiple testing using the Bonferroni method.

Data analysis and visualisation was done using R and ‘Tidyverse’ suite of R packages (48).

## RESULTS

### Recombinases as genomic markers for MGE identification

Different MGE types are associated with specific recombinase families and subfamilies. To be able to capture all known MGE types, we utilised the subfamily information of five major recombinase families, namely tyrosine (Tyr), serine (Ser), HUH, DDE (including DEDD and PDDEXK related) and cas1 solo (Cas) recombinases (23,49,50). For accurate recombinase subfamily detection and to account for the considerable recombinase sequence diversity, we obtained recombinase HMMs at the subfamily level, since it offers (i) the necessary resolution to distinguish the cellular recombinases from those associated with MGEs, as has recently been demonstrated for tyrosine recombinases (28) and (ii) allows to disentangle instances where recombinases from one family appear in distinct MGEs; for example, specific DDE recombinase subfamilies are distinctively found only in IS elements, others in transposons or phages (51). Using 42 calibrated Hidden Markov Models (HMM) from Pfam (16), 20 recombinase subfamilies from literature (28) and six newly created HMM profiles (see Methods and Supplementary Figure S1 and S2A) we covered to the best of our knowledge all known recombinase (sub)families reported in prokaryotic MGEs (Figure 1A and Supplementary Table S1). We did not consider phages that do not integrate in the host genome and hence might lack a recombinase as their ability to modify host genomes via horizontal transfer of genes is very low (52). Our set of 68 marker HMMs also includes four host (i.e. cellular) tyrosine recombinase subfamilies that we use for comparisons and quality control.

Applying these HMMs to proGenomes2 (22), a database of 84,022 high quality genomes from 10179 species (see Supplementary Table S2) selected from NCBI, we identified 2972237 recombinases (for family and subfamily distribution see Supplementary Figure S2B and C). As accurate ‘seeding’ of MGEs is essential for follow up analyses, we compared our recombinase set to the functional annotations within the proGenomes2 (22) database, which are based on EggNOG5 orthologous families (17). Our recombinase detection approach recovered 97% (2352235) of the annotated recombinases. Almost all of the 3% (60214) missed recombinases were resolvases (serine recombinases) that are likely involved in genomic inversions (54) rather than in MGE propagation, and were hence not considered when building supervised MGE-associated serine recombinase HMMs. To compare to other functional annotation pipelines, we also analysed 4.85 million RefSeq (55) proteins from representative genomes, and recovered 97.6% (31963) of the RefSeq-annotated recombinases. Of the missed 2.3% (756), the vast majority contained domains of unknown functions (DUFs) and transposon-associated proteins without any sequence similarity to known recombinases, as confirmed by an independent Pfam search against the entire database of protein domains.

Furthermore, our HMM-approach revealed additional ∼690k recombinases in proGenomes2 (22) that were not annotated by EggNOG (Supplementary Figure S3A). To test the accuracy of these predictions, we first performed reciprocal searches of the proteins against the 68 HMMs. We found that 96.3% of them agreed at the recombinase subfamily level and 3.1% at the family level. The remaining 0.6% (3924) of newly predicted recombinases did not match any domain in the entire Pfam database and could be either highly diverged sequences or a small fraction of false positives. In addition, we determined and compared the domain composition of the 2.2 million (known) recombinases already annotated by EggNOG and the ∼690k newly predicted (novel) recombinases and used their associated domains as independent validation for correct recombinase prediction (Supplementary Figure S3B and C). 37% of the novel recombinases (compared to 45% of the known ones) contained non-catalytic domains that are typically associated with recombinases, including arm-binding domains associated with Tyr recombinase subfamilies (28,56), Zn ribbon recombinase domains associated with Ser recombinase family (35,57) and DNA-binding domains of DDE recombinase (58). These domains showed significant association with the predicted subfamilies, as shown in Supplementary Figure S3B and C, endorsing our recombinase predictions. Fewer than 1% of the novel recombinases (4939) did not share non-catalytic associated domains with the proteins in the known fraction (but neither do some of the annotated recombinases). The comparison of domain associations within the known and novel fractions of recombinases thus validated the specificity of the annotations and also revealed a vast unexplored underlying amount of distinct functional domains that are significantly associated with recombinases (for an example see Figure 1C and Supplementary Figure S3B and C), hinting at specific functionality.

Finally, we screened for the presence of potential active site residues within all the identified recombinase gene products (see Methods and Supplementary Figure S3D and E). On average, 96% of both the annotated and the newly predicted recombinases contained at least one recombinase catalytic domain with a putative active site (Supplementary Figure S3E), providing independent validation for our recombinase detection framework. In several cases, non-catalytic domains resided next to catalytic ones, suggesting that they have either retained DNA-binding capability or provide other beneficial functional aspects (Supplementary Figure S3D and see Figure 1C for an example).

Taken together, all benchmarks on predicted recombinases revealed a very high accuracy, with almost no known false negatives and maximal 0.6% false positives. Thus, in addition to achieving a much higher functional resolution than existing recombinase annotations, we can confidently report an almost 30% increase of high accuracy recombinase predictions, compared to the widely used EggNOG (17) annotations.

### MGE boundary estimates and MGE type assignments

Since different MGE types are not always uniquely determined by the recombinase subfamily, contextual information on the MGE type-specific functionalities is needed, which, in turn, requires a good and consistent estimation of MGE boundaries.

As different MGE types differ tremendously in length, and methods for boundary detection are limited to a few MGE subtypes (7,10,59,60), we employed a universal approach to estimate MGE boundaries. We utilised the pangenomes annotated in proGenomes2 (22) and focused on a subset of ∼76k genomes from ∼3k species that also qualified as ‘high quality’ when using CheckM (61), i.e. ≥90% completeness and ≤5% contamination see Supplementary Table S2. These ∼76k genomes also had to have at least two sequenced strains per species to be able to define core and accessory genes (3), as by definition, MGEs should be successive stretches of accessory genes including at least one recombinase, thus providing an upper limit for the MGE length.

As few close conspecific strains might not provide sufficient discriminative power for distinguishing accessory from core genes, we tested the dependence of MGE length prediction on the number of conspecific strains. Predicted MGE length slightly decreased with an increasing number of conspecific strains, but the differences were minor (Supplementary Figure S4A and B) and all estimates were in the expected size range per MGE type (7,9–11,62). Thus, we continued with the dataset of 76k genomes containing ∼2.6 million recombinases and defined the upper-limits of MGE boundaries by concatenating accessory genes (defined based on their presence in <95% of the conspecific strains in a species) around the identified marker recombinases until the nearest core gene (present in at least 95% of the conspecific strains) or contig boundary was encountered (see Materials and Methods for detailed considerations). This region was labelled as recombinase island for further reference. Of the ∼2.6 million MGE recombinases detected in the 76k genomes, 91% were accessory genes. The remaining 9% of the recombinases were classified as core genes, potentially due to the presence of the respective MGEs in closely related conspecific strains or due to MGE domestication (59,63). Hence, recombinase islands for these recombinases were also considered in our analysis, after further processing (see Materials and Methods). In contrast to MGE recombinases, 63% of the 125k identified cellular recombinases were core genes. The annotation of the remaining 37% as accessory genes may reflect the hijacking of cellular recombinases by mobile elements (28,64) or their occasional absence across bacterial genomes as proposed previously (65). Since MGE boundary estimates can also be hampered by nested MGE regions with different types or copies of recombinases (66–69), ∼434K out of the ∼1.8 million recombinase islands containing more than one recombinase were flagged as nested (cf. Figure 1C), suggesting a potential co-occurrence of MGEs and an increased MGE length. Nested MGEs thus lead to potential inflation of MGE boundary estimates of the individual MGEs within the nested element.

We next utilised contextual information using the genes within the estimated MGE boundaries as they might encode characteristic MGE type-specific functionality. For instance, genes encoding the conjugation machinery and phage structural genes are hallmarks of plasmids and phages, respectively. We used such known MGE type functional markers to classify MGEs (see Materials and Methods), thus implicitly confirming the recombinase prediction and disentangling MGE types that share recombinase subfamilies (Supplementary Figure S5A and Supplementary Table S1). As there was still ambiguity for a few MGE types, we defined six operational MGE categories that can be mapped to MGE types: (i) transposable elements, that is ISs and transposons (contain the same recombinase subfamily markers with no other MGE-type specific genes), (ii) phages (phage recombinase subfamily markers with phage structural genes within the predicted MGE boundaries), (iii) phage-like elements (phage recombinase subfamily markers without supporting, recognizable phage structural genes), (iv) conjugative elements (CE) that is plasmids and ICEs (containing recombinase subfamily markers and conjugation machinery genes, mapped using TXSScan (34)); (v) mobility islands (MI) (unclassified phage/CE recombinase subfamily markers which cannot be resolved due to the absence of neighbouring phage structural genes and genes encoding for secretion systems or due to the simultaneous presence of both of them) and (vi) integrons, containing specific recombinase subfamily markers.

### Quantification of MGEs across the prokaryotic tree of life

**Figure 2.**
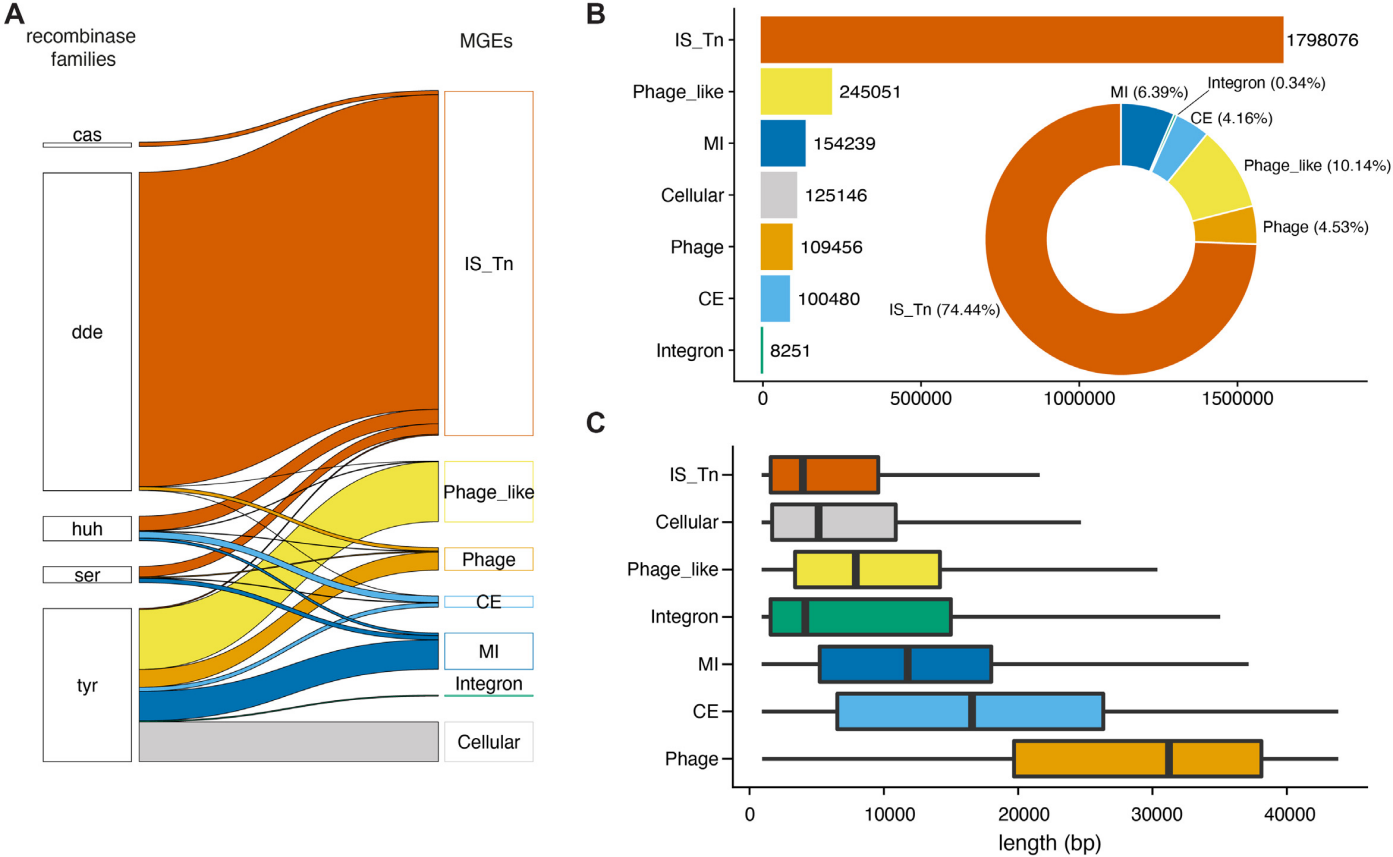
A comprehensive prokaryotic MGE census (**A**) Contribution of each of the five major recombinase families to the six different MGE categories as well as to cellular recombinases. (**B**) Number of MGEs per category (barplot) and percentage of categories (donut chart) in 76k genomes from 3k species using the workflow described in Figure 1. (**C**) Predicted lengths of non-nested MGEs in base pairs. The whiskers span from the 10th to the 90th percentile

When grouping the identified subset of ∼2.6 million MGE recombinases into the 6 MGE categories (Figure 2A), the 1.7 million transposable elements (including at least 235k putative IS elements, Supplementary Figure 5B) clearly stood out, followed by 109k Phages and 245k Phage-like elements, 154k Mobility Islands, 100k Conjugative Elements and 8k Integrons (Figure 2B). Excluding nested recombinase islands with more than one MGE, the MGE length distributions were all within the expected length distributions of the respective types (7–11) (Figure 2C).

**Figure 3.**
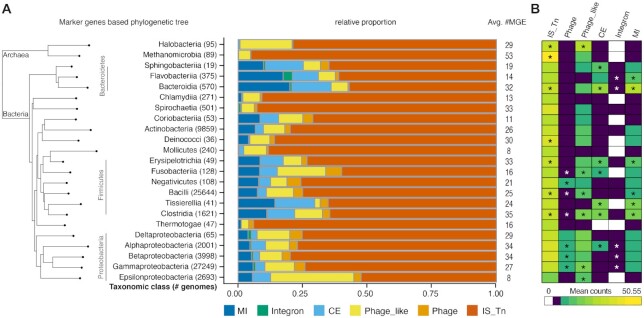
Taxonomic distribution of MGEs. (**A**) Prevalence of MGE categories and dominance of transposable elements across taxonomic classes (with at least 10 genomes), sorted by taxonomic marker gene-based phylogeny (38). (**B**) Association of MGE categories (average MGE counts per species) with different taxonomic classes (Wilcoxon rank-sum test, * indicates *P*-value < 0.05 after Bonferroni correction).

To compare our unifying MGE category predictions with dedicated MGE type-specific tools, we predicted the two most-studied MGE types, transposable elements and phages, in sampled representative genomes, one from each of the 23 different taxonomic classes depicted in Figure 3A (see Materials and Methods and Supplementary Figure S5C). For transposable elements, we used ISEScan (43) and despite its conceptually different approach as well as its known error rates, we observed an almost complete overlap with our predictions (Supplementary Figure S5C). For phages, we used PHASTER (42) and predicted ∼7 times more phages than PHASTER in our set of tested genomes (75 versus 11 with an overlap of 5, where all the non-overlapping PHASTER predictions had low scores and only one contained a recombinase, Supplementary Figure S5C). Thus, we implicitly expand phage predictions in proGenomes2 (22), which is not surprising as phage diversity is vastly underestimated (70). However, our predicted MGE boundaries, in particular for transposable elements and phages, were longer compared to ISEScan and PHASTER predictions, respectively (median length difference ∼7 and ∼13 kb, respectively). These observed differences in length can be attributed to the feature of our method of predicting upper limits of MGE boundaries and to the fusion of nested MGEs into longer stretches (see Supplementary Table S3 and methods for description of MGE boundaries for nested elements).

We next analysed the taxonomic distribution of predicted MGEs and observed that they are all prevalent across the prokaryotic phylogeny (Figure 3). IS elements and transposons dominated every taxonomic class of both Archaea and Bacteria (Figure 3A). On average, a genome harboured ∼7 IS elements or transposons (Supplementary Figure S5D). However, the number of MGEs per species varied considerably across different taxonomic classes, with Mollicutes and Epsilonbacteria harbouring the fewest (<10 MGEs on average) and Methanomicrobia the most (>50 MGEs on average; Figure 3A). After correcting for the dependency of MGE occurrences on genome size (Spearman rho = 0.31, *P*-value ≤ 2.2e−16 and Supplementary Figure S5E) and only considering taxonomic classes with at least 10 genomes to reduce stochastic effects, we found that Proteobacteria and Firmicutes were clearly enriched in phages, while Bacteroidetes and Firmicutes were enriched in conjugative elements and mobility islands (Figure 3B).

### Pervasive MGE-mediated HGT between taxa and habitats

**Figure 4.**
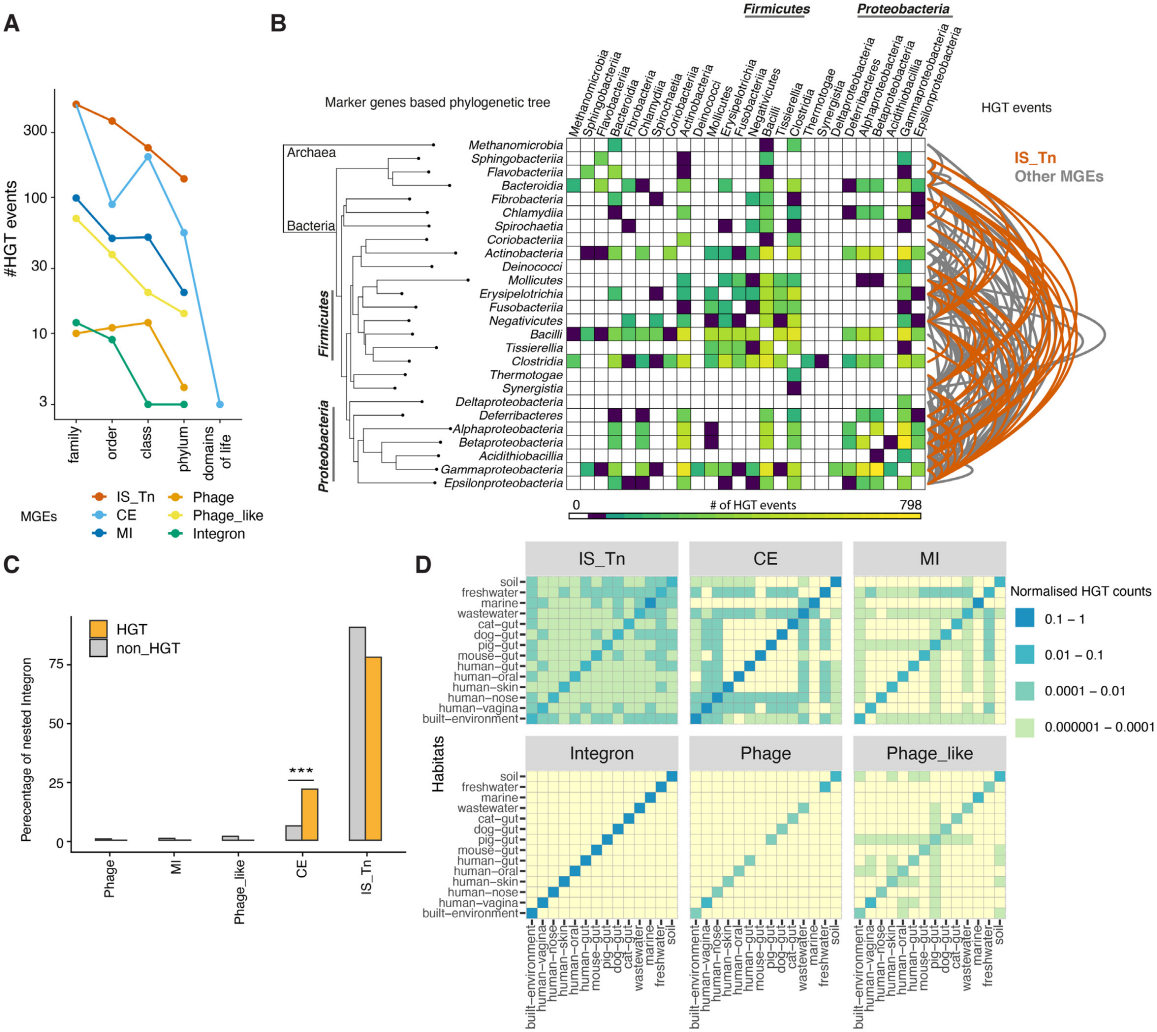
MGE-mediated Horizontal Gene Transfer (HGT). (**A**) Occurrences of recent MGE-mediated HGT events decrease considerably with taxonomic distance; (**B**) Overview of MGE-mediated HGT events across a phylogenetic tree (based on phylogenetic marker genes) of taxonomic classes. The heatmap quantifies the HGT events (coloured according to the legend at the bottom) between taxonomic classes and arcs indicate the contribution of transposable elements (in red) over other MGE categories (in grey). (**C**) Nesting analysis of integrons with different MGE categories shows their significant nesting with CE- Conjugative elements (Fisher's odds ratio 4.3, *P*-value = 2.9e−86) in HGT subset compared to all nested occurrences. (**D**) Heatmaps illustrating the promiscuous horizontal transfer of transposable elements across habitats compared to other MGE categories. All MGE categories show high within habitat MGE dynamics (diagonals) compared to between habitat.

To compare the dynamics and propagation of the different MGE categories with respect to their potential impact on host genome evolution, we systematically studied the abundance of horizontal gene transfer (HGT) events. To unambiguously quantify HGT events, we defined an HGT if an MGE-associated recombinase shared more than 95% sequence identity (a value that usually represents intraspecies variation (71,72)) across distant clades (i.e. between taxonomic families up to domains of life) (see Materials and Methods). Our strict criteria lead to inclusion of only relatively recent transfers spanning ∼1.1–2.3 Mya (assuming a substitution rate of 2.2 × 10^–8^ to 4.5 × 10^–9^ per bp per year (73–75)). Furthermore, as most of the HGT is assumed to occur between closely related species or even within species (76–78), supported by our data (Figure 4A) and as we only study a likely biased fraction of existing genomes, the total number of inwards and outwards-directed HGTs is much larger. Nevertheless, we identified as many as 6536 recent HGT events at the family level or above (3701 of these containing identical sequences) implying a remarkable HGT frequency between distant taxa, also because we studied only a tiny fraction of the prokaryotic diversity that came from diverse habitats, locations and studies.

We detected multiple HGT events in each of our MGE categories, albeit with varying frequencies. For all the MGE categories, we observed a decline in HGT frequency from the more fine-grained taxonomic levels to HGT between domains of life (Figure 4A). When only considering HGT events at taxonomic class level or beyond, transposable elements clearly dominated accounting for 50.9% of the distant HGT events (Figure 4A and Supplementary Figure S6A and 6B). This was unexpected as transposable elements lack active inter-cellular transfer capabilities, as opposed to phages and conjugative elements. As transposable elements can hitchhike with phages or conjugative elements (66), and to better understand how MGEs of the different categories interact and disperse in general, we next analysed their nestedness (as stated in Materials and Methods). Among the ∼434k recombinase islands containing nested elements that we detected (see above), integrons, which lack both intra and inter-cellular transfer mechanisms (74–76,79), were most nested with other MGE categories (63%), whereas we found only 17% nesting of transposable elements. When we compared the association of integrons and transposable elements with other MGE categories in the HGT subset over all of their nested occurrences, we found them to be significantly enriched with conjugative elements and phage-like elements respectively in the HGT subset (*P* < 0.05, one sided Fisher's exact test, Figure 4C and Supplementary Figure S6C). For the most recent HGTs (i.e. identical sequences) we found integrons and transposable elements to be enriched in nesting with conjugative elements (*P* < 0.05, one sided Fisher's exact test, Supplementary Figure S6C).

MGE-mediated HGT events occurred in each bacterial taxonomic clade at class level, indicating that MGEs shape the gene pools across the prokaryotic tree of life (Supplementary Figure S6A) considerably. The most frequent HGT events were observed within and between the phyla Firmicutes and Proteobacteria (Figure 4B) and were mediated by most MGE categories. In turn, HGT events between archaea and bacteria (i.e. different domains of life) were restricted to conjugative elements (Supplementary Figure S6A). We found evidence for the simultaneous presence of entire MGEs up to 13kb across domains of life (e.g. *Bacteroides ovatus* SAMN05192581 and *Methanosarcina mazei* SAMN02708976 Figure 1C; see supplementary text for technical validations) providing compelling evidence for recent HGT of entire MGEs.

As HGT needs physical proximity, we further explored MGE-mediated HGT within and between habitats (see Materials and Methods). We observed extensive within-habitat HGT for almost all MGE categories (Figure 4D), whereas the frequency of between-habitat transfers was considerably lower. When considering HGT events between habitats, transposable elements were the most promiscuous and were transferred indiscriminately across all 14 operational habitat categories (see methods, Figure 4D and Supplementary Figure S6D). Conjugative elements showed transfer across half of these habitats, while phages and integrons did not show any horizontal transfer between habitats (Figure 4D).

Taken together, MGE-mediated HGT appears very frequently and, while enriched across similar taxa and within habitats, it occurs also between different domains of life and distinct habitats. Due to their general abundance and ability to hitchhike on conjugative elements, transposable elements are the dominating HGT facilitators.

### MGE-mediated cargo gene dispersal: antibiotic resistance as a case in point

**Figure 5.**
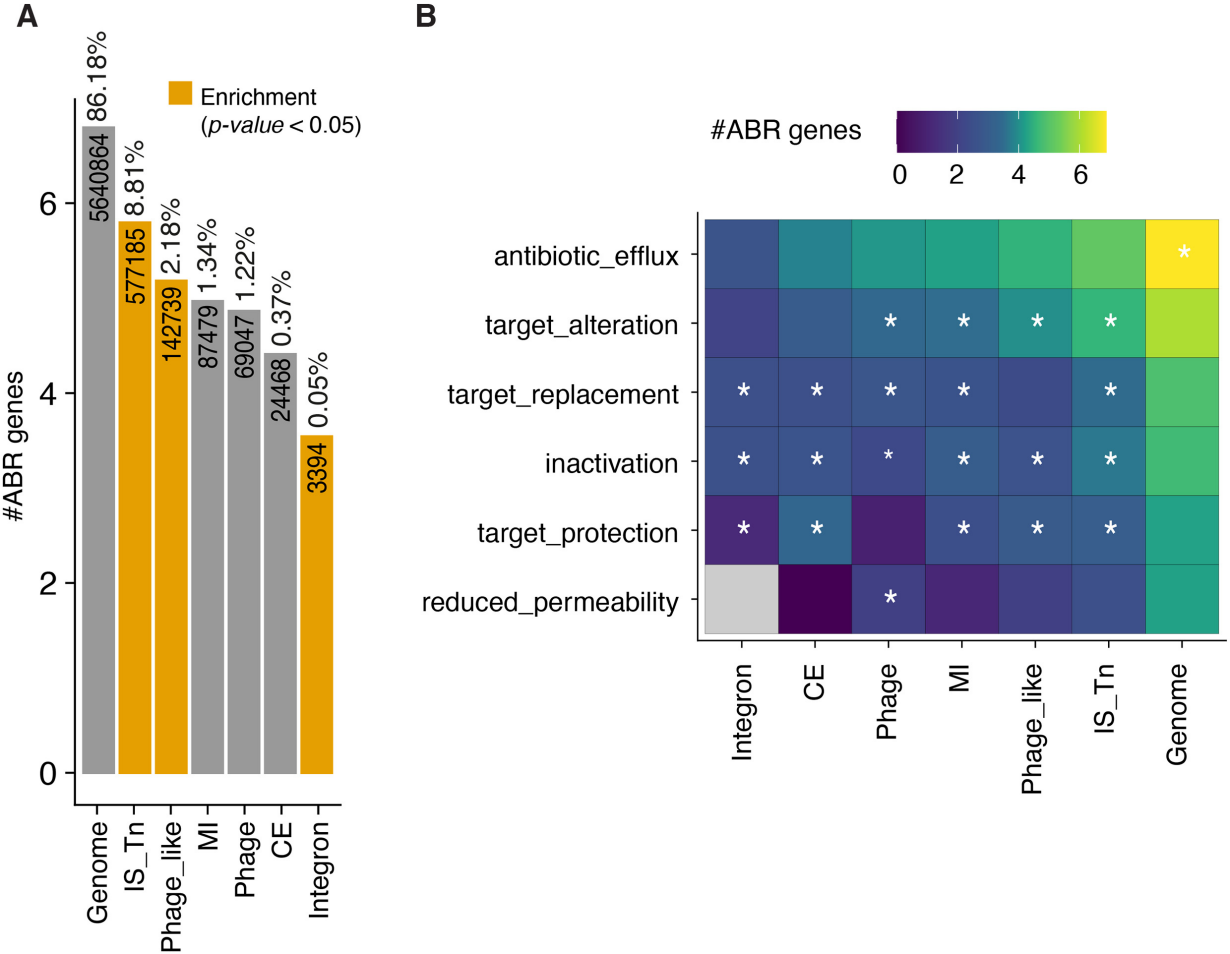
Antibiotic Resistance Genes (ARGs) carrying potential of MGEs. (**A**) Analysis of MGEs (per category) that carry ARGs show enrichment of ARG in transposable elements compared with other genomics regions, indicating transposable elements as major MGE associated ARG carriers. (**B**) Association of antibiotic resistance mechanisms with different MGE categories. Asterisks indicate significant enrichment according to one sided Fisher's exact test (*P*-value < 0.05 after Bonferroni correction).

Our computational framework enables the characterization of MGE cargo genes including genes of significant medical importance, such as antibiotic resistance genes (ARGs). As ARGs pose a significant threat to human lives and there is an urgent need for understanding the mechanisms of their global dispersal, we quantified ARGs within the estimated boundaries of each annotated MGE (see Materials and Methods). On average, between 29% of all MGEs analysed in this study carried an ARG, compared to 16% of genomic (non-MGE) regions (Supplementary Figure S7A). Of the HGT events described in the previous section, only 15% involved antibiotic resistance genes (ARGs) as cargo, which is a large underestimate due to our strict operational HGT definition and might explain the decrease compared to the overall number. Yet, among the MGE categories, ARGs were significantly enriched in transposable elements (Fisher's odds ratio 1.5, *P*-value = 0), integrons (Fisher's odds ratio 1.6, *P*-value = 2.7e−148) and phage-like elements (Fisher's odds ratio 1.23, *P*-value = 0) (Figure 5A). To confirm that these patterns are not driven by sampling bias of host- and disease-associated prokaryotic genomes in publicly available databases, we looked for enrichment of ARGs on MGEs exclusively in non-host associated bacteria (soil, aquatic, food habitats) in our dataset and found similar patterns of ARG enrichment with different MGE categories (Supplementary Figure S7B).

We further investigated the antibiotic resistance spreading capabilities of MGEs by analysing the enrichment of known major resistance mechanisms. Although antibiotic efflux effectors, which includes ABC transporters and other efflux pumps, constitute the majority of proteins encoded by ARGs across all MGEs, they are not globally enriched over the Genome (Figure 5B). Although different MGE categories were enriched in diverse resistance mechanisms, we found ARGs from all major resistance mechanisms can spread via MGEs (Figure 5B) and be transmitted across distant taxa and habitats.

Antibiotic resistance is a medical threat in human-associated microbiota, but the respective ARGs also prevail in complex communities in terrestrial and other habitats (80). We found that MGEs in all habitats carry ARGs, and a comparison between MGE categories revealed integrons and transposable elements as the primary carriers (Supplementary Figure S7C).

Adaptive traits, like antibiotic resistance, can promote the maintenance of MGEs within host genomes and can also manifest through regulatory functional domains within the recombinases themselves. In particular, recombinases can contain domains regulating heavy metal resistance genes, known to often co-occur with ARGs (81,82). For example, we observed that MerR domains (83) are significantly enriched in certain serine recombinase occurring in transposable elements (Supplementary Figure S3C). Furthermore, we also found recombinases of ARG-carrying MGEs with ‘addiction’ domains that confer immunity against antibiotics/toxins involved in bacterial competition. For instance, relaxases of most Streptococci carry ‘Streptin-immun’ domains, conferring immunity against their own antibiotic, Streptin, which is used to weed out competing bacteria in the environment (53) (Figure 1C).

Thus, the high fraction of ARGs or ARG-associated domains in MGEs and their HGT potential is striking and underpins the possible extent of ARG transfer between diverse taxa and habitats.

## DISCUSSION

Our consistent annotation and analysis of mechanistically and functionally diverse MGE types provides a first comprehensive overview of the prokaryotic MGE landscape with insights into their phylogenetic and environmental prevalence and dispersal patterns. The respective results have been integrated into our MGE resource, enabling a detailed and yet exhaustive MGE annotation in 76k high quality genomes from >3000 prokaryotic species from diverse phyla. Based on our data, MGEs cover as many as 19.3% of the accessory genome (and 13% of the entire genome) on average, more than previously suggested in prokaryotic genomes based on an *Escherichia coli* analysis alone (84,85), with differing contributions from six MGE categories (cf. proMGE.embl.de). Although, discoveries of hitherto unknown MGE types and variants (86) may increase this fraction in the future, it might be counterbalanced by an expected slight decrease in estimated MGE sizes as the addition of diverse species from distinct habitats should also improve the already high accuracy of the annotations. For example, more conspecific strains for a given species and improved quality of complete genomes will improve the accuracy of MGE boundary estimates.

Our comparative survey of MGEs across ∼275 diverse taxonomic prokaryotic families revealed the overall taxa and habitat wide dominance of transposable elements over other MGE types. We found a number of MGE types associated with different taxa or habitats. For example, Proteobacteria, showed a clade specific enrichment of phage and phage-like elements, and habitats like the human gut were enriched in conjugative elements and mobility islands, in particular in the common gut bacterial phyla of Firmicutes and Bacteroidetes (Figure 3B). This implies that both habitat (Figure 4D) and taxonomy influence the occurrence and prevalence of most MGE types in a species.

Our conceptually novel approach leads to novel biological insights in various ways. First, the recombinase collection uncovered the unexplored adaptive potential of recombinases by (i) domain acquisition and revealed a large variability of DNA binding domains, which potentially influence MGE insertion capabilities or influence the efficiency of DNA cleavage like in the case of arm-binding (AB) domain of tyrosine recombinases (87) (Supplementary Figure S3) and (ii) non-catalytic recombinase domains, residing next to catalytic ones, suggesting their potential for functional divergence (Figure 1C for an example).

Second, recombinase markers and MGE-type independent element boundary estimates allowed us to capture MGE interactions of different types through nesting information of ∼1.2 million recombinases. Our quantification of nesting provides mechanistic insights into the dispersal of the majority of integrons and 17% of the transposable elements via hitchhiking with other MGEs. The observed enrichment of transposable element nestedness in identified HGT events for identical sequences (35% over 17% overall), occurring mostly with conjugative elements (Supplementary Figure S6), provides a partial mechanistic explanation for their dispersal. Since a large fraction of the transposable elements were not nested, we hypothesize jumping off of transposable element to another genomic location upon entry and/or the existence of alternate spreading scenarios, e.g. via independent transfer during conjugation, natural transformation (uptake and incorporation of foreign DNA into the genome (88)), or hitherto unknown mechanisms.

Third, we illustrated by an analysis of ARG cargo, how our framework can be used to track both presence and MGE-mediated transfer of ARG across distant taxa and different habitats, illuminating transposable elements as major antibiotic resistance gene carriers. However, transposable elements are known to lack intercellular transfer mechanisms and as described above rely on MGE nesting interactions with other MGE categories for their horizontal transfer, implying the necessity to consider presence of all MGEs together to monitor spread of resistance. Thus, in the future mitigation of MGE mediated spread of ARGs will depend on the ability to predict acquisition and spread of ARGs by diverse MGEs, which requires understanding and analysis of ARG-MGE co-occurrence patterns in prokaryotic genomes (89). Our resource proMGE provides this necessary underlying data to facilitate future research on the spread of multi-drug resistance. However, while making such interpretations, it is important to consider a few caveats of the MGE predictions in our database (i) MGE boundaries represent upper limits of genomic regions that harbour one or more MGEs of same or different types; (ii) all proteins within the MGE boundaries might not be sufficiently annotated, so that some of our gene context-based approaches for MGE category assignment might overlook them; (iii) beyond the recombinase marker gene, phage structural genes for phages and conjugation machinery genes for conjugative elements other gene features may not be annotated and or well-defined for all predicted MGEs.

The impacts of MGEs on the global dispersal of adaptive molecular functions go far beyond antimicrobial resistance though; in fact, we found that ARGs constitute less than 1% of all MGE cargo genes, this is a very small fraction even considering that we estimate only the upper limits of MGE boundaries. Other examples of cargo include genes encoding for defence systems (90), virulence and metabolic factors (2) and bacterial addiction systems (91,92), which might hold clues for understanding the role of MGEs and their interactions in niche expansion of the host bacterium. With more genomes and metagenomes from diverse habitats to be sequenced in the future, our framework and the associated resource should enable a much higher resolution view on MGE-mediated gene transfer across species and habitats, thus increasing our knowledge of their roles in species adaptation and evolution. These advancements may implicitly also enable actions on preventing the MGE-mediated spread of unwanted functionality, such as antibiotic resistance.

## DATA AVAILABILITY

The data generated in this study is available on the developed resource proMGE: http://promge.embl.de/. The 68 recombinase profile HMMs are available on http://smart.embl-heidelberg.de/ and these can be searched using profile HMM names provided in Supplementary Table S1 under subfamily. Additionally, MGE recombinases can be annotated using query protein sequences on proMGE resource page under ‘Annotate’ tab. Files and scripts for data analysis and generation of figures can be found at https://git.embl.de/khedkar/promge

## Supplementary Material

gkac163_Supplemental_Files

## Notes

Present address: Falk Hildebrand, Gut Microbes and Health, Quadram Institute Bioscience, Norwich, Norfolk, UK.

Present address: Falk Hildebrand, Digital Biology, Earlham Institute, Norwich, Norfolk, UK.

Present address: Mechthild Luetge, Institute of Immunobiology, Kantonsspital St. Gallen, 9007 St. Gallen, Switzerland.
