# Supplementary material for: Landscape of mobile genetic elements and their antibiotic resistance cargo in prokaryotic genomes
Source: Nucleic Acids Res. 2022 Mar 22;50(6):3155–68. doi: 10.1093/nar/gkac163 (PMC8989519; doi:10.1093/nar/gkac163)
Supplement: gkac163_Supplemental_Files [file gkac163_supplemental_files.zip › proMGE_revision_supplementary_data.pdf]

Landscape of mobile genetic elements and their antibiotic resistance cargo in prokaryotic genomes

Supriya Khedkar[1], Georgy Smyshlyaev[1,2], Ivica Letunic[3], Oleksandr M. Maistrenko[1], Luis Pedro Coelho[4], Askarbek Orakov[1], Sofia K. Forslund[1,5,6,7], Falk Hildebrand[1,8,9], Mechthild Luetge[1,10], Thomas S. B. Schmidt[1], Orsolya Barabas[1,2], Peer Bork[1,5,11,12*]

[1]European Molecular Biology Laboratory, Structural and Computational Biology Unit, 69117 Heidelberg, Germany

[2]Department of Molecular Biology, University of Geneva, 1211 Geneva, Switzerland

[3]Biobyte solutions GmbH, Bothestr 142, 69117 Heidelberg, Germany

[4]Institute of Science and Technology for Brain-Inspired Intelligence, Fudan University, Shanghai 200433, China

[5]Max Delbrück Centre for Molecular Medicine, Berlin, Germany

[6]Experimental and Clinical Research Center, Charité-Universitätsmedizin and Max-Delbrück Center, Berlin, Germany

[7]Charité – Universitätsmedizin Berlin, Berlin, Germany

[8]Present address: Gut Microbes and Health, Quadram Institute Bioscience, Norwich, Norfolk, UK

[9]Present address: Digital Biology, Earlham Institute, Norwich, Norfolk, UK

[10]Present address: Institute of Immunobiology, Kantonsspital St. Gallen, 9007 St. Gallen, Switzerland

[11]Department of Bioinformatics, Biocenter, University of Würzburg, Würzburg, Germany

[12]Yonsei Frontier Lab (YFL), Yonsei University, Seoul 03722, South Korea

*Correspondence: peer.bork@embl.org

Supplementary figure legends

Supplementary Figure 1. HMM building and calibration workflow

Workflow for building recombinase subfamily profile HMMs (see Methods)

Supplementary Figure 2. Evaluation and abundance of recombinase subfamily profile HMMs

A. Evaluation statistic: recall, specificity, f.p.r (false positive rate), accuracy and m.c.c (mathews correlation coefficient) of the performance of the built recombinase subfamily profile HMMs on the training dataset; B. Abundance of DDE recombinase based on recombinase family specific counts of the five major recombinase families; C. Recombinase subfamily counts for each of the five major families represented in panel B.

Supplementary Figure 3. Validation of recombinase annotations

A. Comparable counts of recombinase predictions by EggNOG and this study; B and C. Panels corresponding to five major recombinase families representing large repertoire of diverse domains significantly associated with recombinases; D. Majority of recombinase subfamilies showing the presence of the putative active site in the known and novel fraction either in a single recombinase domain of a protein or in one of the recombinase domains in a multi-domain protein; E. Major proportion of proteins across all recombinase subfamilies showing presence of putative active site residue.

Supplementary Figure 4. Evaluation of pangenome features

A. Box plots comparing the percentage of accessory genes within a species pangenome as a function of number of strains within a species; B. Average length (in base pairs) of MGE categories coloured according to the number of conspecific strains within a species (numbers in boxes indicate the total number of MGEs in each category).

Supplementary Figure 5. MGE assignment and quantification

A. Prokaryotic MGE disentanglement workflow representing MGE category determination strategy for recombinase subfamilies shared by different MGE types; B. Black bar showing the small contribution of putative IS element counts (transposable elements with a single transposase gene) to total transposable element numbers (same as Figure 2A with separate IS elements counts); C. Venn diagram showing the substantial overlap in transposable element predictions from ISEScan and this study, and expansion of phage predictions by this study compared to phage predictions by PHASTER; D. High average counts of transposable

elements per genome compared to other MGE categories. The whiskers span from the 10th to the 90th percentile; E. Positive correlation between number of MGEs in a genome and proteome size (Spearman's rho = 0.31, $p < 2.2e-16$).

Supplementary Figure 6. MGE-mediated Horizontal Gene Transfer (HGT)

A. Marker gene based prokaryotic phylogenetic tree with coloured arcs representing MGE mediated HGT events across taxonomic classes (same as Figure 4A with arcs coloured according to MGE categories); B. High transposable elements mediated distant HGT events compared to other MGE categories; C. Enrichment of horizontally transferred IS_Tn with phage_like elements (HGT calls at 95% sequence identity) and conjugative elements (HGT calls at 100% sequence identity see methods) implicating mechanism of inter-cellular transfer of identical sequences IS_Tn via hitchhiking; D. Heatmaps illustrating the promiscuous horizontal transfer of transposable elements across habitats compared to other MGE categories. All MGE categories show high within habitat MGE dynamics (diagonals) compared to between habitat (same as Figure 4D but with absolute counts).

Supplementary Figure 7. Potential of MGEs to carry ARG across habitats

A. Distribution of normalised Antibiotic Resistance Genes (ARG) counts per 1000 genes for each MGE category compared to the (non-mobile) Genome. On average 29% of all MGEs carry at least one ARG. The fraction for each MGE category is indicated on the boxplot; B. Analysis of MGEs (per category) that carry ARGs in non-host associated habitats (soil, aquatic and food habitat) shows enrichment of ARG in transposable elements compared with other genomics regions, indicating transposable elements as major MGE associated ARG carriers; C. Distribution of ARGs per 1000 genes by MGE category and separated by habitat shows high fractions of ARGs in transposable elements and integrons across all habitats.

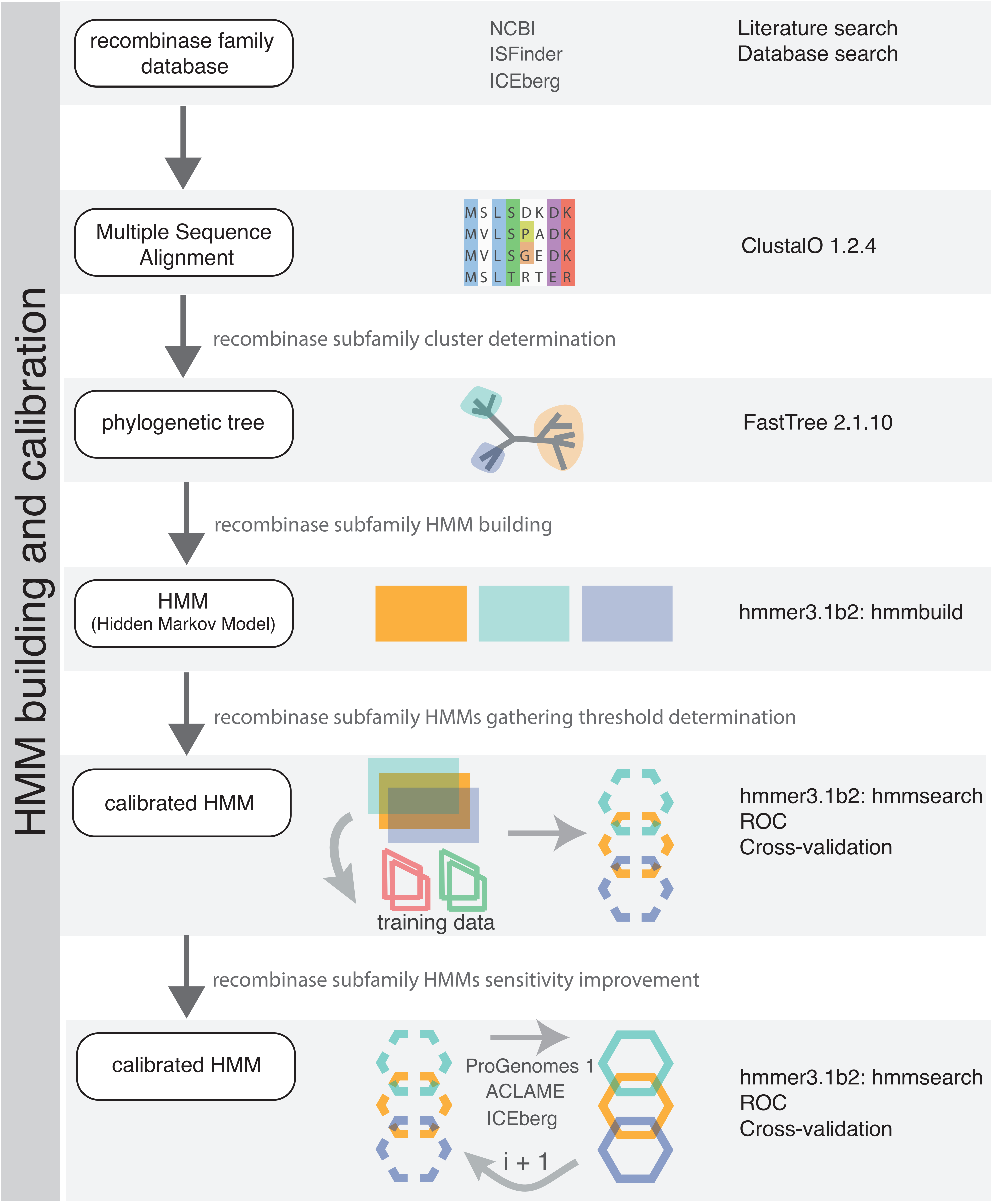

HMM building and calibration
recombinase family database
NCBI
ISFinder
ICEberg
Literature search
Database search
Multiple Sequence Alignment
M S L S D K D K
M V L S P A D K
M V L S G E D K
M S L T R T E R
ClustalO 1.2.4
recombinase subfamily cluster determination
phylogenetic tree
FastTree 2.1.10
recombinase subfamily HMM building
HMM
(Hidden Markov Model)
hmmer3.1b2: hmmbuild
recombinase subfamily HMMs gathering threshold determination
calibrated HMM
training data
hmmer3.1b2: hmmsearch
ROC
Cross-validation
recombinase subfamily HMMs sensitivity improvement
calibrated HMM
ProGenomes 1
ACLAME
ICEberg
i + 1
hmmer3.1b2: hmmsearch
ROC
Cross-validation

Figure S1

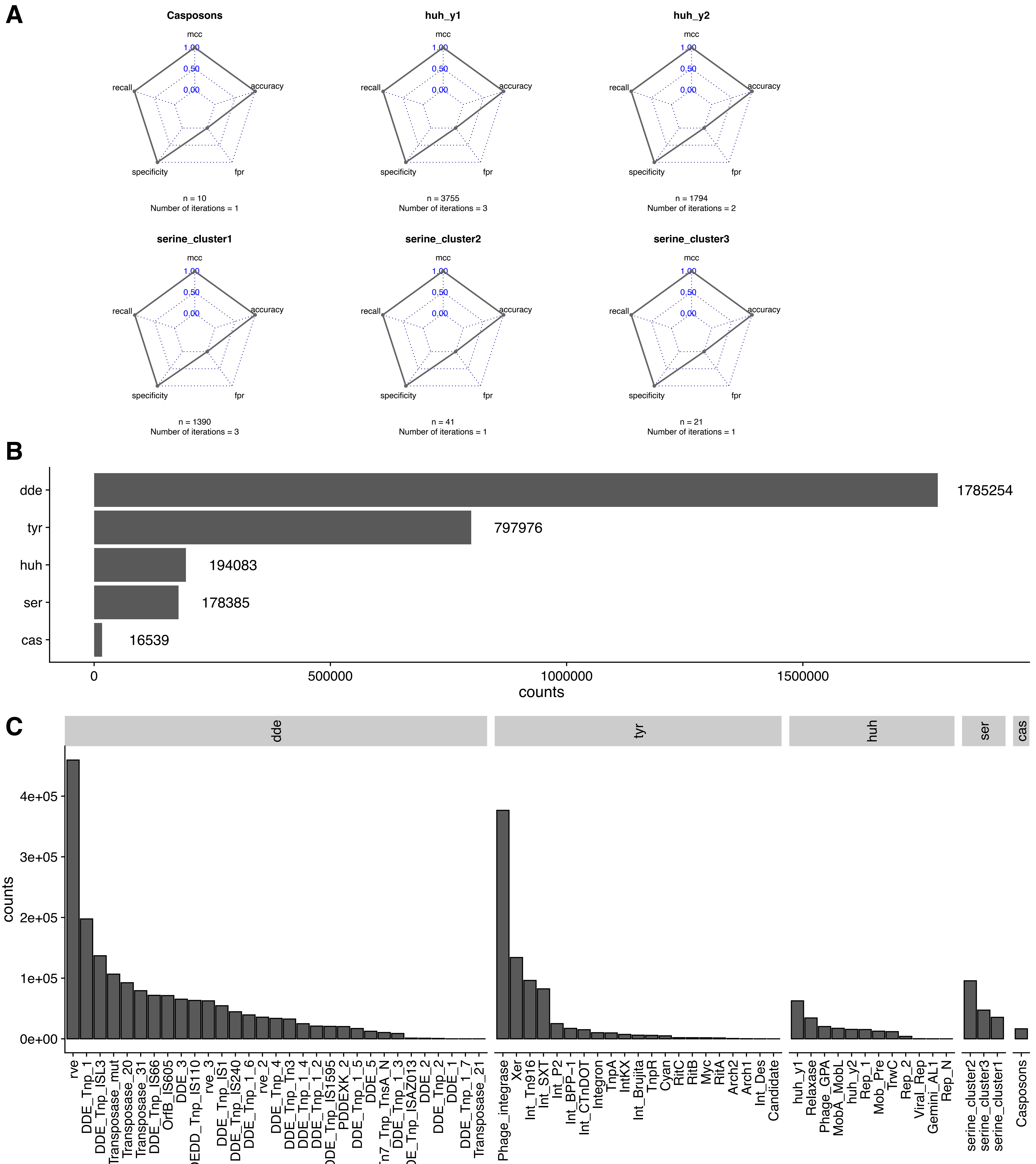
A
Casposons
huh_y1
huh_y2
serine_cluster1
serine_cluster2
serine_cluster3
mcc
accuracy
fpr
specificity
recall
1.00
0.50
0.00
n = 10
Number of iterations = 1
n = 3755
Number of iterations = 3
n = 1794
Number of iterations = 2
n = 1390
Number of iterations = 3
n = 41
Number of iterations = 1
n = 21
Number of iterations = 1
B
dde
tyr
huh
ser
cas
1785254
797976
194083
178385
16539
0
500000
1000000
1500000
counts
C
dde
tyr
huh
ser
cas
counts
0e+00
1e+05
2e+05
3e+05
4e+05
rve
DDE_Tnp_1
DDE_Tnp_ISL3
Transposase_mut
Transposase_20
Transposase_31
DDE_Tnp_IS66
OrfB_IS605
DDE_3
DEDD_Tnp_IS110
rve_3
DDE_Tnp_IS1
DDE_Tnp_IS240
DDE_Tnp_1_6
rve_2
DDE_Tnp_4
DDE_Tnp_Tn3
DDE_Tnp_1_4
DDE_Tnp_1_2
DDE_Tnp_IS1595
PDDEXK_2
DDE_Tnp_1_5
DDE_5
Tn7_Tnp_TnsA_N
DDE_Tnp_1_3
DDE_Tnp_ISAZ013
DDE_2
DDE_Tnp_2
DDE_1
DDE_Tnp_1_7
Transposase_21
Phage_integrase
Xer
Int_Tn916
Int_SXT
Int_P2
Int_BPP-1
Int_CTnDOT
Integron
TnpA
IntKX
Int_Brujita
TnpR
Cyan
RitC
RitB
Myc
RitA
Arch2
Arch1
Int_Des
Candidate
huh_y1
Relaxase
Phage_GPA
MobA_MobL
huh_y2
Rep_1
Mob_Pre
TrwC
Rep_2
Viral_Rep
Gemini_AL1
Rep_N
serine_cluster2
serine_cluster3
serine_cluster1
Casposons

Figure S2

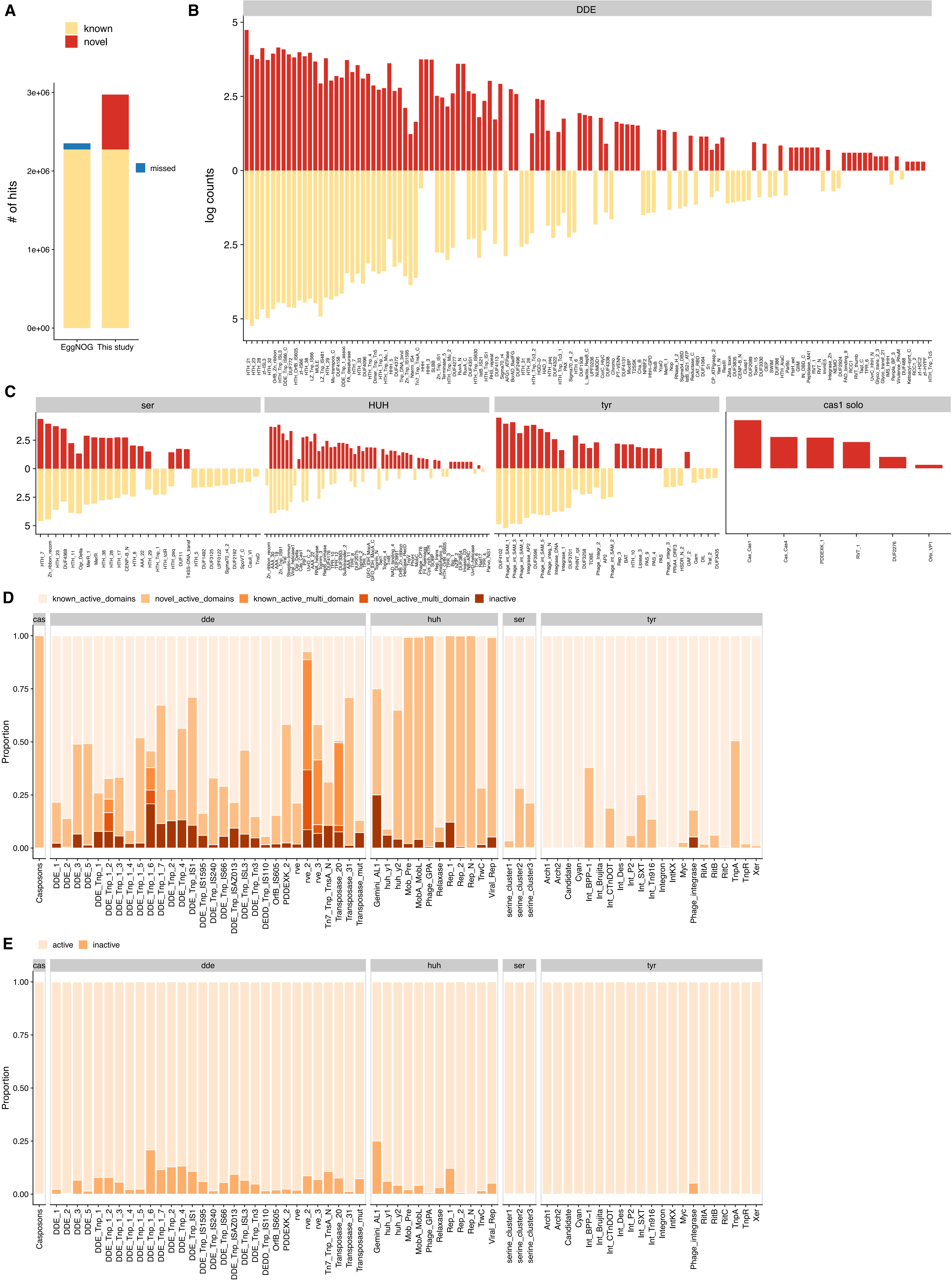
A
known
novel
missed
# of hits
EggNOG
This study
B
DDE
log counts
C
ser
HUH
tyr
cas1 solo
D
known_active_domains
novel_active_domains
known_active_multi_domain
novel_active_multi_domain
inactive
cas
dde
huh
ser
tyr
Proportion
Casposons
DDE_1
DDE_2
DDE_3
DDE_5
DDE_Tnp_1
DDE_Tnp_1_2
DDE_Tnp_1_3
DDE_Tnp_1_4
DDE_Tnp_1_5
DDE_Tnp_1_6
DDE_Tnp_1_7
DDE_Tnp_2
DDE_Tnp_4
DDE_Tnp_IS1
DDE_Tnp_IS1595
DDE_Tnp_IS240
DDE_Tnp_IS66
DDE_Tnp_ISAZ013
DDE_Tnp_ISL3
DDE_Tnp_Tn3
DEDD_Tnp_IS110
OrfB_IS605
PDDEXK_2
rve
rve_2
rve_3
Tn7_Tnp_TnsA_N
Transposase_20
Transposase_31
Transposase_mut
Gemini_AL1
huh_y1
huh_y2
Mob_Pre
MobA_MobL
Phage_GPA
Relaxase
Rep_1
Rep_2
Rep_N
TrwC
Viral_Rep
serine_cluster1
serine_cluster2
serine_cluster3
Arch1
Arch2
Candidate
Cyan
Int_BPP-1
Int_Brujita
Int_CTnDOT
Int_Des
Int_P2
Int_SXT
Int_Tn916
Integron
IntKX
Myc
Phage_integrase
RitA
RitB
RitC
TnpA
TnpR
Xer
E
active
inactive
recombinase sub-families

Figure S3

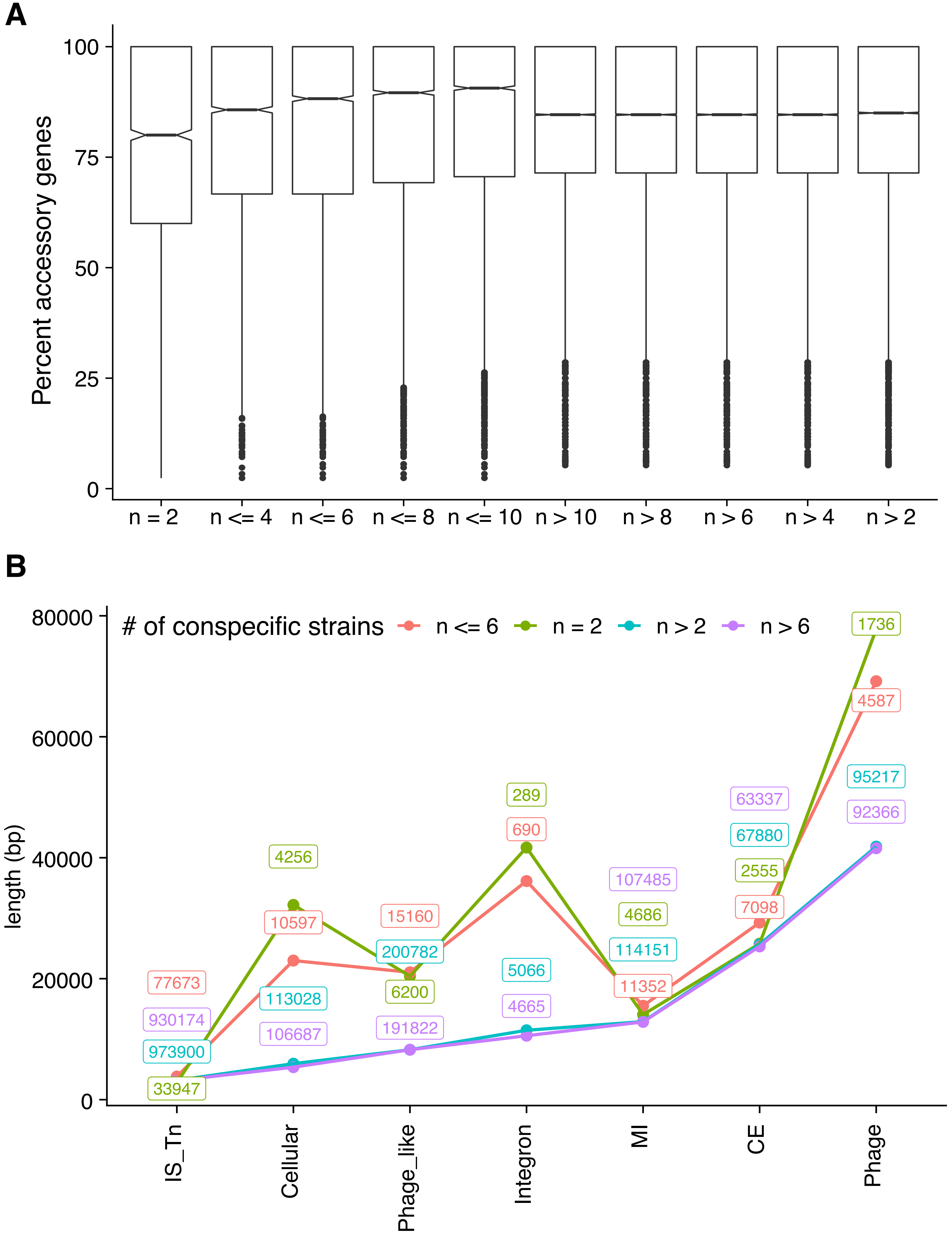

A
Percent accessory genes
100
75
50
25
0
n = 2
n <= 4
n <= 6
n <= 8
n <= 10
n > 10
n > 8
n > 6
n > 4
n > 2
B
# of conspecific strains
n <= 6
n = 2
n > 2
n > 6
length (bp)
80000
60000
40000
20000
0
IS_Tn
Cellular
Phage_like
Integron
MI
CE
Phage
77673
930174
973900
33947
4256
10597
113028
106687
15160
200782
6200
191822
289
690
5066
4665
107485
4686
114151
11352
63337
67880
2555
7098
1736
4587
95217
92366

Figure S4

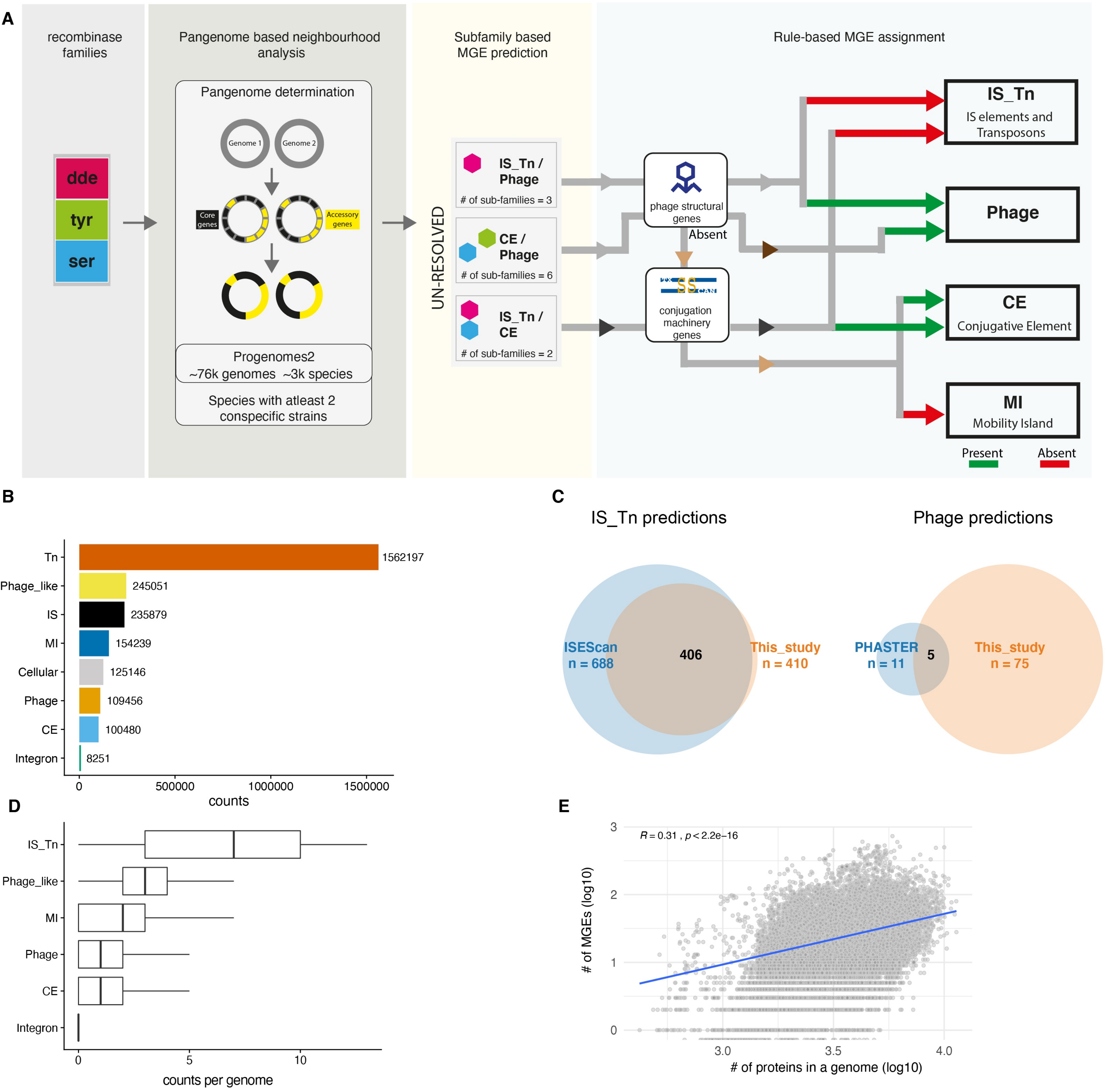
A
recombinase families
dde
tyr
ser
Pangenome based neighbourhood analysis
Pangenome determination
Genome 1
Genome 2
Core genes
Accessory genes
Progenomes2
~76k genomes ~3k species
Species with atleast 2 conspecific strains
Subfamily based MGE prediction
UN-RESOLVED
IS_Tn / Phage
# of sub-families = 3
CE / Phage
# of sub-families = 6
IS_Tn / CE
# of sub-families = 2
Rule-based MGE assignment
phage structural genes
Absent
conjugation machinery genes
IS_Tn
IS elements and Transposons
Phage
CE
Conjugative Element
MI
Mobility Island
Present
Absent
B
Tn
Phage_like
IS
MI
Cellular
Phage
CE
Integron
1562197
245051
235879
154239
125146
109456
100480
8251
0
500000
1000000
1500000
counts
C
IS_Tn predictions
ISEScan n = 688
406
This_study n = 410
Phage predictions
PHASTER n = 11
5
This_study n = 75
D
IS_Tn
Phage_like
MI
Phage
CE
Integron
0
5
10
counts per genome
E
R = 0.31 , p < 2.2e−16
# of MGEs (log10)
0
1
2
3
3.0
3.5
4.0
# of proteins in a genome (log10)

Figure S5

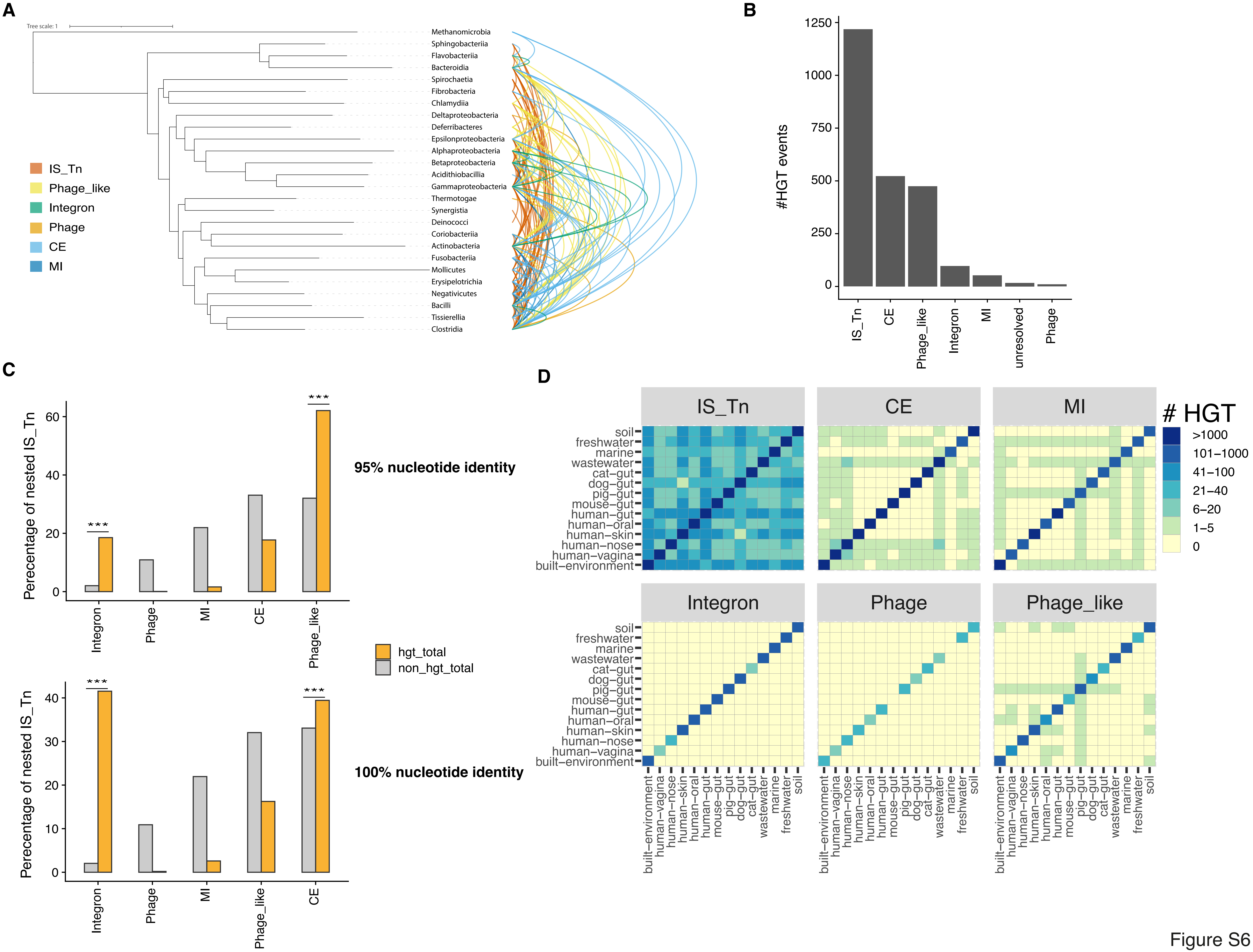
A
Tree scale: 1
Methanomicrobia
Sphingobacteriia
Flavobacteriia
Bacteroidia
Spirochaetia
Fibrobacteria
Chlamydiia
Deltaproteobacteria
Deferribacteres
Epsilonproteobacteria
Alphaproteobacteria
Betaproteobacteria
Acidithiobacillia
Gammaproteobacteria
Thermotogae
Synergistia
Deinococci
Coriobacteriia
Actinobacteria
Fusobacteriia
Mollicutes
Erysipelotrichia
Negativicutes
Bacilli
Tissierellia
Clostridia
IS_Tn
Phage_like
Integron
Phage
CE
MI
B
#HGT events
1250
1000
750
500
250
0
IS_Tn
CE
Phage_like
Integron
MI
unresolved
Phage
C
Perecentage of nested IS_Tn
60
40
20
0
***
***
Integron
Phage
MI
CE
Phage_like
95% nucleotide identity
hgt_total
non_hgt_total
Perecentage of nested IS_Tn
40
30
20
10
0
***
***
Integron
Phage
MI
Phage_like
CE
100% nucleotide identity
D
IS_Tn
CE
MI
Integron
Phage
Phage_like
soil
freshwater
marine
wastewater
cat−gut
dog−gut
pig−gut
mouse−gut
human−gut
human−oral
human−skin
human−nose
human−vagina
built−environment
HGT
>1000
101−1000
41−100
21−40
6−20
1−5
0

Figure S6

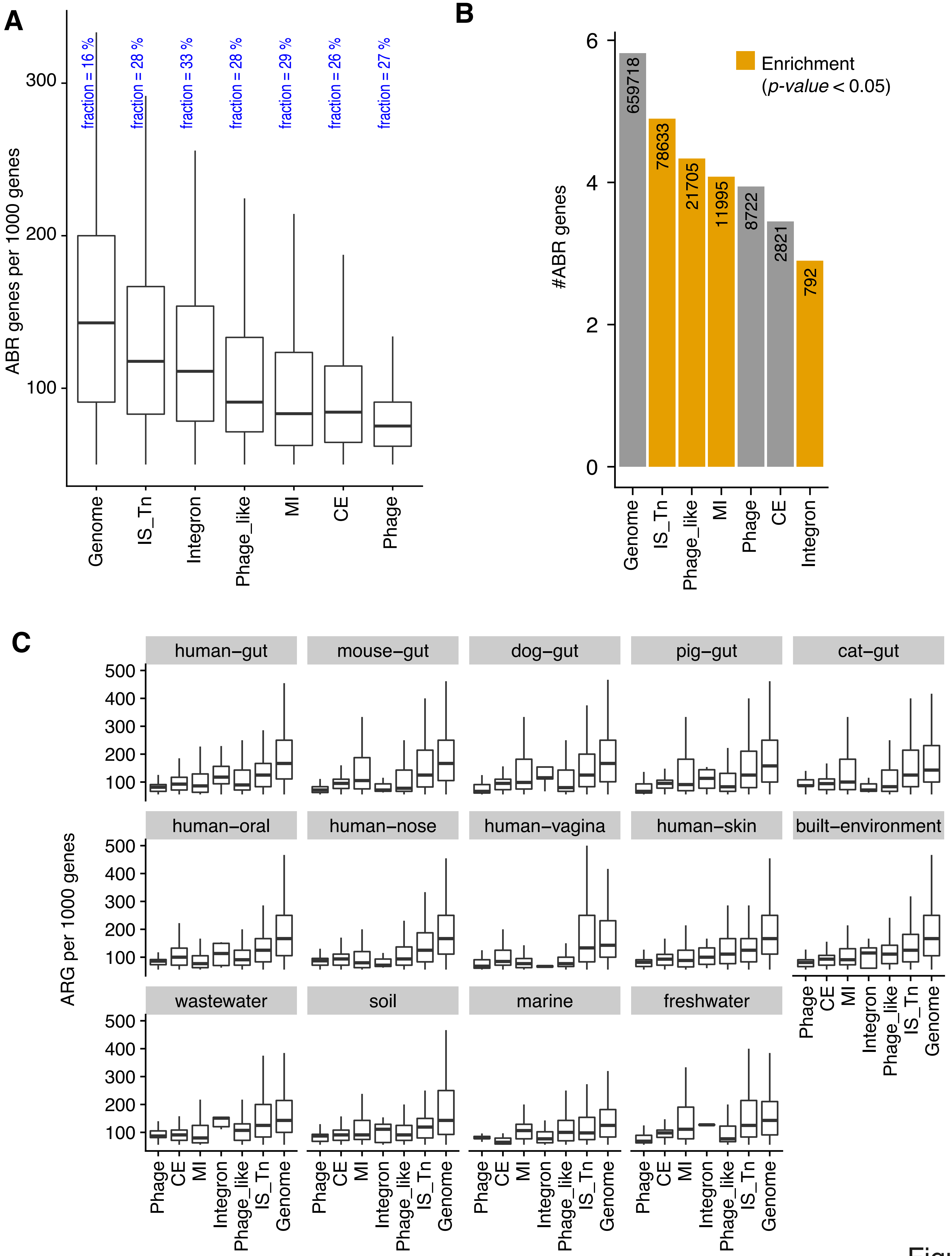
A
ABR genes per 1000 genes
fraction = 16 %
fraction = 28 %
fraction = 33 %
fraction = 28 %
fraction = 29 %
fraction = 26 %
fraction = 27 %
Genome
IS_Tn
Integron
Phage_like
MI
CE
Phage
B
#ABR genes
Enrichment
(p-value < 0.05)
659718
78633
21705
11995
8722
2821
792
Genome
IS_Tn
Phage_like
MI
Phage
CE
Integron
C
ARG per 1000 genes
human−gut
mouse−gut
dog−gut
pig−gut
cat−gut
human−oral
human−nose
human−vagina
human−skin
built−environment
wastewater
soil
marine
freshwater
Phage
CE
MI
Integron
Phage_like
IS_Tn
Genome

Figure S7
