# Supplementary material for: Landscape of mobile genetic elements and their antibiotic resistance cargo in prokaryotic genomes
Source: Nucleic Acids Res. 2022 Mar 22;50(6):3155–68. doi: 10.1093/nar/gkac163 (PMC8989519; doi:10.1093/nar/gkac163)
Supplement: gkac163_Supplemental_Files [file gkac163_supplemental_files.zip › proMGE_Supplementary_table_text.docx]

**Landscape of mobile genetic elements and their antibiotic resistance cargo in prokaryotic genomes**

Supriya Khedkar^1^, Georgy Smyshlyaev^1,2^, Ivica Letunic^3^, Oleksandr M. Maistrenko^1^, Luis Pedro Coelho^4^, Askarbek Orakov^1^, Sofia K. Forslund^1,5,6,7^, Falk Hildebrand^1,8,9^, Mechthild Luetge^1,10^, Thomas S. B. Schmidt^1^, Orsolya Barabas^1,2^, Peer Bork^1,5,11,12*^

^1^European Molecular Biology Laboratory, Structural and Computational Biology Unit, 69117 Heidelberg, Germany

^2^Department of Molecular Biology, University of Geneva, 1211 Geneva, Switzerland

^3^Biobyte solutions GmbH, Bothestr 142, 69117 Heidelberg, Germany

^4^Institute of Science and Technology for Brain-Inspired Intelligence, Fudan University, Shanghai 200433, China

^5^Max Delbrück Centre for Molecular Medicine, Berlin, Germany

^6^Experimental and Clinical Research Center, Charité-Universitätsmedizin and Max-Delbrück Center, Berlin, Germany

^7^Charité – Universitätsmedizin Berlin, Berlin, Germany

^8^Present address: Gut Microbes and Health, Quadram Institute Bioscience, Norwich, Norfolk, UK

^9^Present address: Digital Biology, Earlham Institute, Norwich, Norfolk, UK

^10^Present address: Institute of Immunobiology, Kantonsspital St. Gallen, 9007 St. Gallen, Switzerland

^11^Department of Bioinformatics, Biocenter, University of Würzburg, Würzburg, Germany

^12^Yonsei Frontier Lab (YFL), Yonsei University, Seoul 03722, South Korea

*****Correspondence: peer.bork@embl.org

**Supplementary Table Legends**

**Table S1** - Classification of Mobile Genetic Element (MGE) recombinase (sub)families and their association with different MGE categories

**Table S2** - Genome quality and assembly status of prokaryotic genomes used in this study

**Table S3** - Comparison of proMGE-ISEscan and proMGE-PHASTER MGE predictions
